# Biosynthesis and Microbial Production of Carminic Acid: From Pathway Elucidation to Synthetic Biology

**DOI:** 10.3390/microorganisms14091869

**Published:** 2026-08-22

**Authors:** Hongyu Li, Jiaqi Liu, Jiashan Lu, Jie Wei, Yuying Bao, Peng Zhang

**Affiliations:** 1 Laboratory of Microbial Resources and Synthetic Biology, Inner Mongolia University, Hohhot 010010, China; 15548834431@163.com (H.L.); xiaoliu020814@163.com (J.L.); lujiashan1017@163.com (J.L.); weijie_211@163.com (J.W.); 2 Inner Mongolia Engineering Technology Research Center of Germplasm Resources Conservation and Utilization, Inner Mongolia University, Hohhot 010010, China

**Keywords:** carminic acid, anthraquinone pigments, synthetic biology, metabolic engineering

## Abstract

Carminic acid (CA) is a high-value natural anthraquinone pigment used in foods, cosmetics, textiles, and pharmaceuticals, but its current industrial supply depends largely on extraction from the scale insect *Dactylopius coccus*, creating constraints in yield, cost, sustainability, and allergen control. This review summarizes recent progress from pathway elucidation to microbial production. We first outline the structure, occurrence, applications, and biosynthetic logic of CA, emphasizing the convergence of type III polyketide assembly with insect-associated tailoring reactions, especially C-glycosylation. We then compare heterologous production strategies in *Escherichia coli*, *Saccharomyces cerevisiae*, *Yarrowia lipolytica*, and *Aspergillus nidulans*, focusing on chassis-specific advantages, bottlenecks, precursor supply, malonyl-CoA engineering, dynamic regulation, enzyme compatibility, compartmentalization, and downstream processing. Structurally related anthraquinone pigments are further discussed to extract broader design principles for pathway diversification and synthetic biology. Finally, we highlight key challenges for industrial translation, including low titers, incomplete enzyme characterization, host–pathway incompatibility, and scalable purification, and propose integrated strategies combining precursor-pathway rewiring, AI-assisted enzyme engineering, biosensor-based regulation, and process optimization to develop competitive microbial cell factories.

## 1. Introduction

Natural colorants have gained unprecedented traction across the food, cosmetic, textile, and pharmaceutical industries, driven by rising consumer demand for clean-label products, stringent regulatory restrictions on synthetic dyes, and growing sustainability awareness [1,2]. Traditional synthetic colorants face multiple safety risks, whereas plant-derived pigments are often compromised by poor structural stability, including high sensitivity to light, heat, oxidation, and pH fluctuations, resulting in rapid fading, low color consistency, and limited industrial applicability [2]. To overcome these limitations, microbial fermentation has emerged as a sustainable and scalable platform for producing a broad spectrum of biocolours, including yellow-to-red carotenoids (e.g., β-carotene and astaxanthin) [3,4], pH-responsive anthocyanins exhibiting red-to-purple-to-blue hues [5,6], violet violacein [7,8], and brilliant blue phycocyanin [9,10,11]. Among these, CA, a high-value natural red anthraquinone pigment, stands out for its exceptional thermal stability, photostability, and biosafety, making it a widely used colorant in food, cosmetics, textiles, and pharmaceuticals [12,13]. Its robust resistance to heat, light, and chemical degradation, coupled with broad pH stability (pH 2–10), effectively addresses the critical shortcomings of conventional plant pigments and underpins its extensive industrial adoption [13,14].

Traditionally, industrial production of CA relies almost exclusively on extraction from the scale insect *D. coccus* Costa. This ancient dye resource has a long cultivation and utilization history traced back to American pre-Columbian civilizations [15,16]. This approach suffers from three major drawbacks: first, supply is strictly constrained by dependence on Opuntia cactus hosts and tropical climatic conditions, with concentrated production regions vulnerable to natural disasters, pests, and diseases [17,18]; second, production costs are prohibitively high, with food- and cosmetic-grade CA priced at 120–240 USD per kilogram, as approximately 70,000 female insects yield only 1 pound of purified product [12,19,20]; third, residual insect-derived proteins carry allergenic risks, and product purity is highly variable due to fluctuations in rearing environments [12,17]. These inherent limitations severely restrict supply stability, increase industrial costs, and compromise product safety and consistency, creating an urgent demand for sustainable, scalable, and controllable alternative production routes [19,21,22].

Synthetic biology has emerged as a transformative solution for the sustainable biosynthesis of complex natural products, enabling the reconstruction of heterologous biosynthetic pathways in genetically tractable microbial hosts [1,23,24,25]. CA biosynthesis is particularly distinctive, as it integrates plant-like type III polyketide assembly with insect-specific tailoring reactions—most notably the unique C-glycosylation step—representing a rare cross-kingdom convergence of biosynthetic logic [14,26,27]. Reconstituting this hybrid pathway demands coordinated optimization of precursor pools, heterologous enzyme folding, subcellular localization and inter-pathway flux balance [28,29,30]. General strategies for microbial cell factory construction have been systematically summarized in multiple landmark reviews, providing universal frameworks for pigment biosynthesis engineering [31,32,33].

To date, no dedicated review has systematically summarized CA biosynthesis and its microbial production. Existing anthraquinone reviews focus predominantly on plant-derived compounds and pharmaceutical activities, while general natural pigment reviews only briefly mention CA without addressing its unique cross-kingdom biosynthetic logic [2]. This review fills three key knowledge gaps: (1) it provides the first end-to-end analysis of CA biosynthetic pathway elucidation and heterologous reconstruction across four microbial chassis; (2) it quantitatively compares the contribution of different engineering strategies and identifies the hierarchical bottlenecks limiting titer improvement; (3) it contextualizes emerging technologies including the NCM pathway, peroxisomal compartmentalization, and AI-assisted enzyme engineering within the specific context of anthraquinone pigment production.

In this review, we provide a comprehensive overview of CA from its structural features, natural occurrence, and industrial applications to the latest advances in its biosynthetic pathway elucidation. We systematically summarize key strategies for microbial production, including chassis selection, precursor pool engineering, metabolic flux redistribution, enzyme engineering, and subcellular compartmentalization. We further place CA in the broader context of structurally related anthraquinones to generalize the principles of pathway diversification and biosynthetic design [34,35]. Finally, we discuss remaining challenges in titer improvement and process scalability, and propose future directions for developing industrially competitive microbial cell factories for CA [36].

## 2. Carminic Acid: Structure, Occurrence and Applications

### 2.1. Chemical Structure

**Figure 1:**
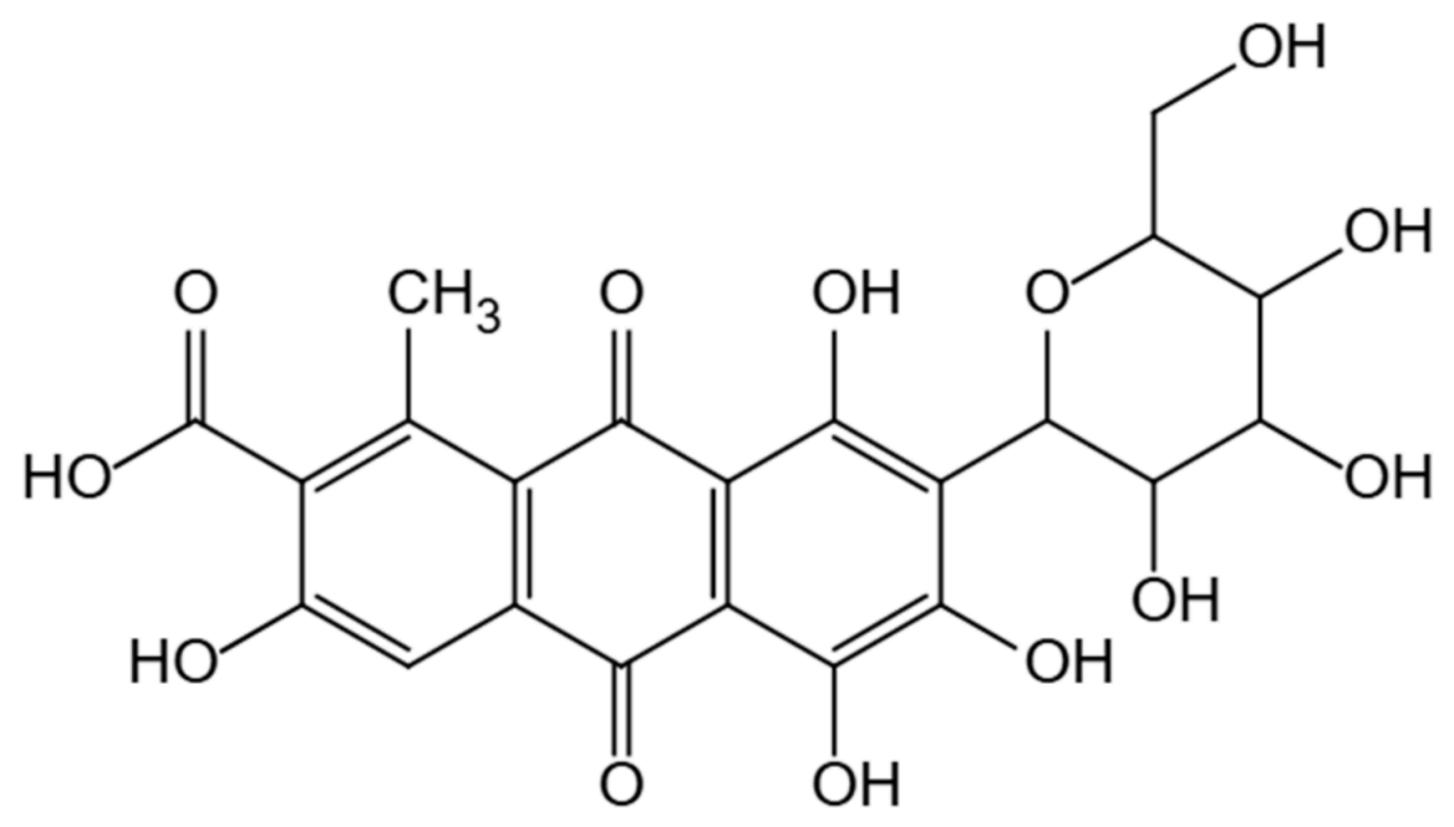
Chemical structure of carminic acid (CA), an anthraquinone C-glycoside natural pigment.

CA is a naturally occurring anthraquinone-derived red pigment that is widely used as a natural colorant in food, cosmetics, and pharmaceutical products. Chemically, it is a C-glycosylated anthraquinone derivative with the molecular formula of C_22_H_20_O_13_ and a molecular weight of 492.39 Da. Structurally, CA consists of an anthraquinone aglycone linked to a glucose moiety through a carbon–carbon (C–glycosidic) bond, a characteristic feature that contributes to its remarkable chemical stability compared with O-glycosides (Figure 1) [12,14].

This structural feature distinguishes CA from many other anthraquinone glycosides that typically contain O-glycosidic bonds [37]. The anthraquinone backbone contributes to the pigment’s high resistance to light, heat, and oxidative degradation, while the attached glucose moiety enhances water solubility and compatibility with diverse matrices [38]. In addition, CA exhibits a broad pH stability range (pH 2–10). The multi-hydroxyl substitution on the tricyclic aromatic ring endows CA pH-responsive color variation, shifting from orange-red under acidic conditions to purple-red under alkaline environments [12]. High-resolution chromatographic and spectroscopic analyses have further clarified the isomer equilibrium of natural CA isolated from cochineal insects [39]. High-performance liquid chromatography combined with mass spectrometry has systematically resolved the structural composition of cochineal anthraquinone mixtures and the equilibrium kinetics of two CA isomers [39,40]. Systematic photophysical characterization of CA and analogous anthraquinone dyes confirms its superior anti-photobleaching performance relative to most plant red pigments [41]. Owing to these physicochemical properties, CA is considered one of the most stable natural red pigments [1,12]. These structural features also play an important role in the biosynthesis and functional properties of CA.

### 2.2. Natural Occurrence

In nature, CA occurs primarily in several species of scale insects, most notably the cochineal insect *D. coccus*. These insects parasitize prickly pear cacti belonging to the genus Opuntia and accumulate large quantities of the pigment within their tissues and hemolymph. The presence of CA gives crushed cochineal insects their characteristic deep red color. Biologically, the compound is believed to function as a chemical defense molecule, protecting the insects from predators and microbial infection [12,14]. Historically, cochineal insects have been cultivated on cactus plantations for the extraction of natural red dyes. After harvesting and drying the insects, CA can be isolated and further processed into cochineal extract or carmine pigment, which has been widely used as a natural colorant for centuries [12].

### 2.3. Industrial Applications

**Figure 2:**
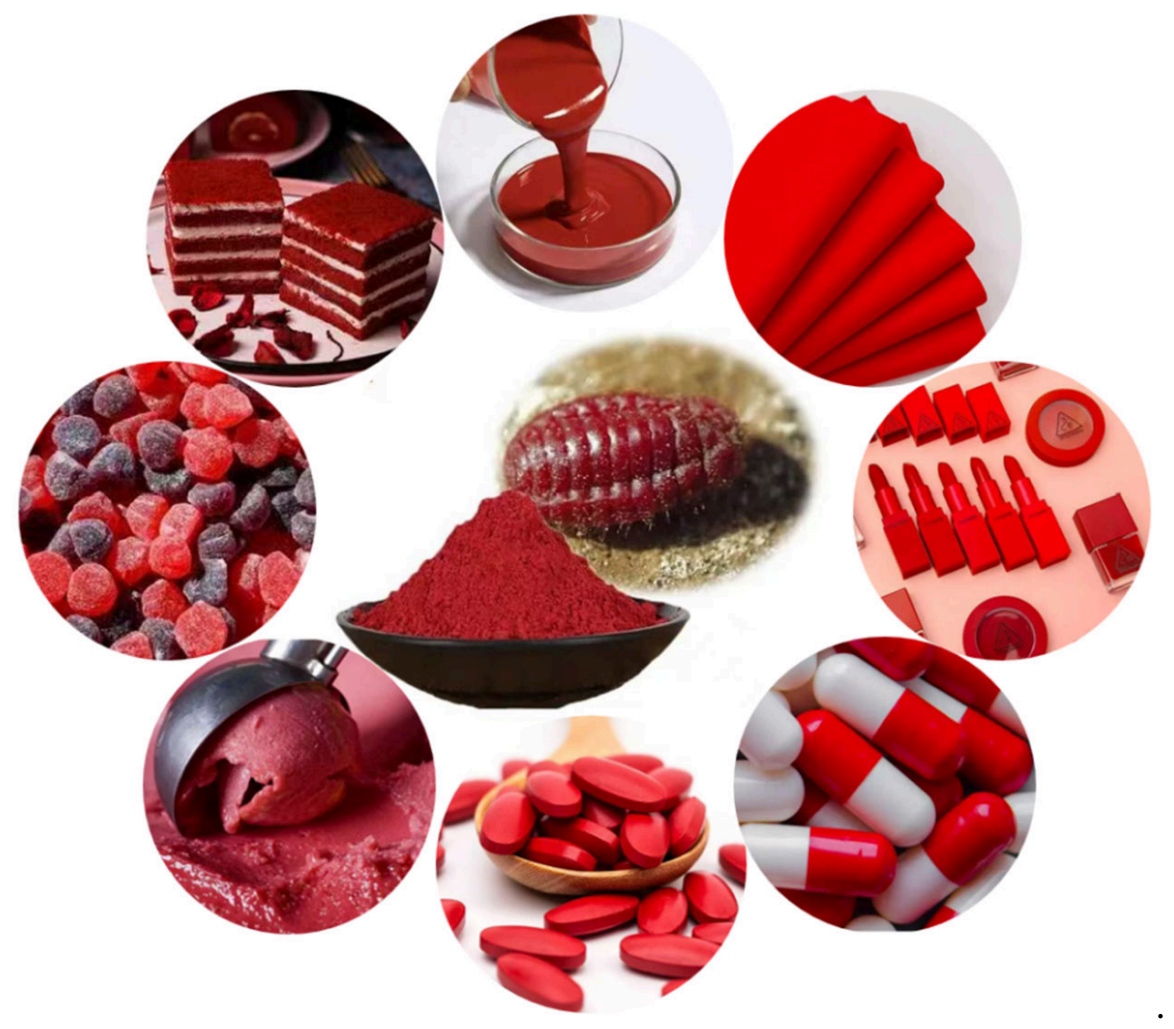
Carminic acid industrial applications. CA is widely used as a natural colorant in the food, cosmetic, pharmaceutical, and textile industries.

Due to its vivid color and exceptional stability, CA has found extensive applications across multiple industries (Figure 2). In the food industry, it is widely used as a natural colorant and is approved as food additive E120 in many countries. It is commonly applied in products such as beverages, confectionery, dairy products, processed meats, and bakery goods to provide stable red or pink hues [1,12]. Beyond coloring, anthraquinone skeletons including CA exhibit moderate antioxidant activity, providing auxiliary pharmaceutical value [42].

In addition to food applications, CA and its aluminum complex pigment, carmine, are also extensively used in the cosmetics industry, particularly in lipsticks, blushes, and other makeup formulations requiring stable red coloration. Furthermore, the pigment is employed in the pharmaceutical industry as a coloring agent in tablets, capsules, and syrups to improve product identification and consumer acceptance [12]. Given the increasing demand for natural colorants and the limitations associated with insect-based production, significant research efforts have been directed toward developing sustainable production strategies for CA, particularly through metabolic engineering and microbial biosynthesis [1,28,29,43].

## 3. Biosynthesis of Carminic Acid

Anthraquinones, including CA, are generally derived from polyketide biosynthetic pathways. In these pathways, iterative condensation of acetyl-CoA and malonyl-CoA units is catalyzed by polyketide synthases (PKSs) to form linear polyketide chains, which subsequently undergo cyclization and tailoring reactions to generate the characteristic anthraquinone scaffold [14,44,45]. Two distinct evolutionary routes produce anthraquinones: the polyketide pathway (in insects, fungi, most plants) and the shikimate-derived pathway (in partial Rubiaceae plants) [34]. Type III PKSs dominate plant and insect polyketide assembly with simple homodimeric architecture independent of ACP carriers [46,47]. Comprehensive reviews have systematically categorized structural and functional differentiation of I/II/III PKS families across microorganisms and plants [47,48,49].

### 3.1. Carminic Acid Biosynthetic Pathway

**Figure 3:**
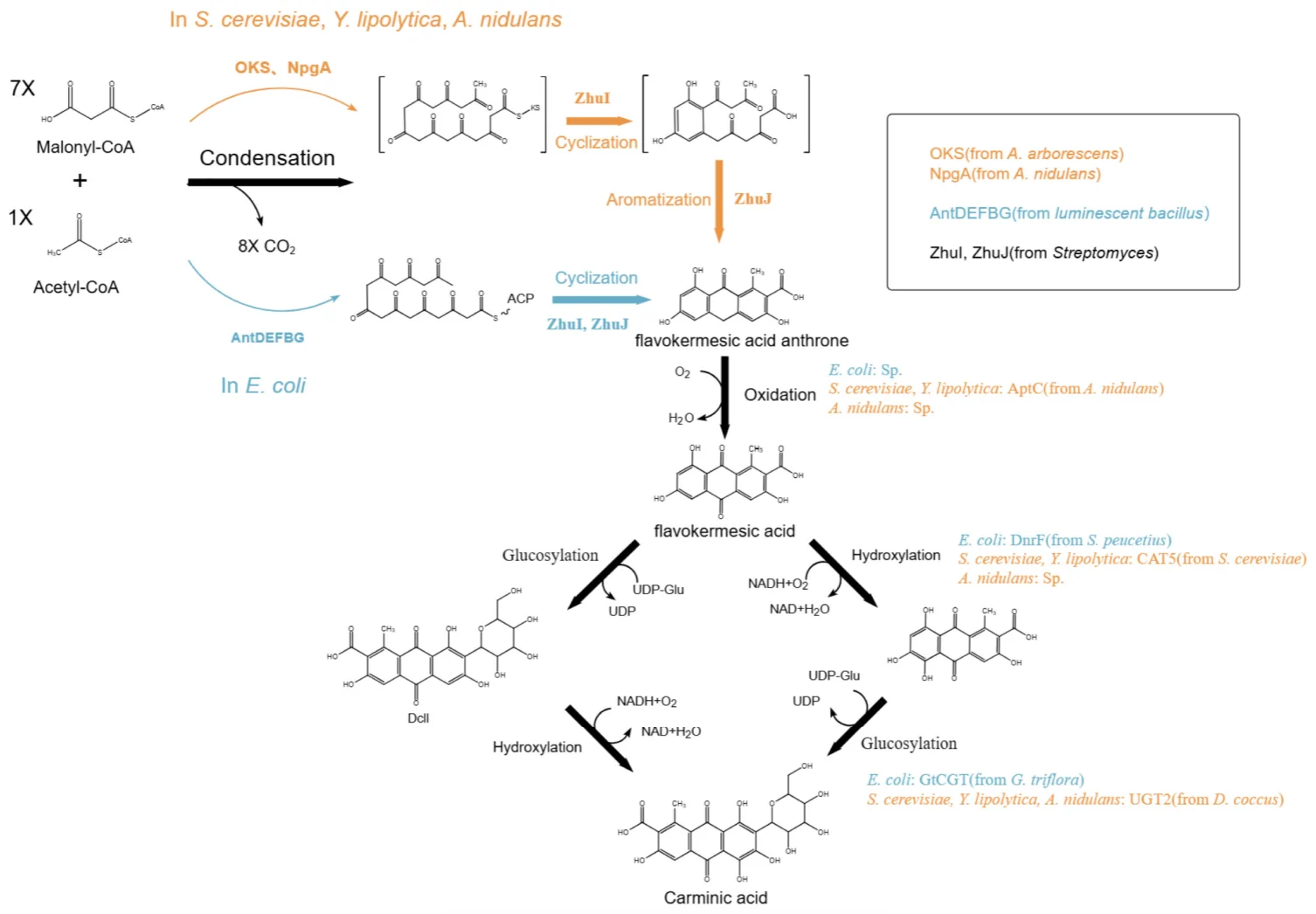
Enzymatic steps and intermediates involved in carminic acid biosynthesis. The pathway comprises three major stages: polyketide chain assembly, anthraquinone scaffold formation, and late-stage functionalization. Octaketide synthase (OKS) catalyzes the formation of the linear octaketide precursor, which undergoes cyclization, aromatization, oxidation, and hydroxylation to generate flavokermesic acid (FK) and kermesic acid (KA). C-glycosylation of these intermediates subsequently leads to the formation of carminic acid (CA). Alternative orders of hydroxylation and C-glycosylation have been proposed for the late-stage pathway.

The biosynthesis of CA originates from acetyl-CoA and malonyl-CoA through a polyketide-based pathway that can be conceptually divided into three functional modules: polyketide chain assembly, anthraquinone scaffold formation, and late-stage functionalization (Figure 3) [14,26]. In the initial step, octaketide synthase (OKS), which comes from *Aloe arborescens*, catalyzes the iterative condensation of one acetyl-CoA starter unit with seven malonyl-CoA extender units to generate a linear octaketide intermediate. Crystallographic structural analysis of minimal octaketide PKS systems has resolved the molecular mechanism of polyketide chain length control [50,51]. This highly reactive intermediate is subsequently directed toward productive biosynthesis through enzyme-controlled cyclization and aromatization, leading to the formation of flavokermesic acid anthrone (FKA). The anthrone intermediate is then oxidized to flavokermesic acid (FK), establishing the core anthraquinone scaffold [26,28]. Downstream of FK, the pathway diverges into two alternative routes leading to CA formation. In one route, FK is first hydroxylated to kermesic acid (KA), followed by C-glycosylation to yield CA. In the alternative route, FK undergoes C-glycosylation to form the intermediate dcII, which is subsequently hydroxylated to produce the final product. This flexibility indicates that hydroxylation and glycosylation are not strictly ordered but represent partially interchangeable late-stage modifications [14,52]. Such modular organization highlights the inherent plasticity of CA biosynthesis and provides a conceptual framework for pathway engineering, where individual modules can be independently optimized or rearranged in heterologous systems.

### 3.2. Key Enzymes for Biosynthesis of Carminic Acid

The key enzymes currently employed or implicated in the heterologous biosynthesis of CA, together with their source organisms, catalytic functions, and levels of experimental validation, are summarized in Table 1.

#### 3.2.1. Octaketide Synthase Mechanism

The formation of the anthraquinone backbone is initiated by octaketide synthase (OKS), a type III polyketide synthase that catalyzes iterative chain elongation. OKS mediates the condensation of one acetyl-CoA molecule with seven malonyl-CoA units to generate a linear octaketide intermediate, which serves as the precursor for subsequent cyclization and aromatization reactions [26,28]. The key characteristics and experimental evidence for AaOKS are summarized in Table 1.

The crystal structure of chalcone synthase (CHS), the archetypal representative of type III PKS, has provided the structural foundation for understanding substrate recognition and chain-length control mechanisms in this enzyme family [53]. Functional studies have demonstrated that plant-derived OKS is sufficient for octaketide backbone assembly. As a type III PKS, OKS operates independently of acyl carrier proteins (ACPs) and directly utilizes CoA-linked substrates, distinguishing it mechanistically from type II PKS systems. This simplified catalytic architecture facilitates heterologous expression and pathway reconstruction across different hosts [26,44]. Site-directed mutagenesis of plant OKS active-site residues has further demonstrated that substrate specificity and product distribution can be modulated by engineering the catalytic pocket [46].

The role of OKS in CA biosynthesis has been experimentally validated through heterologous pathway reconstruction. Expression of *A. arborescens* OKS (AaOKS) enabled the formation of octaketide-derived intermediates in engineered microbial hosts, confirming its ability to supply the required polyketide precursor for downstream anthraquinone formation [26]. However, it should be noted that AaOKS is not an enzyme directly isolated from *D. coccus*. The native insect enzyme responsible for initiating octaketide biosynthesis remains unidentified, and current pathway reconstructions therefore rely on functionally validated surrogate enzymes rather than a fully resolved insect-derived pathway. Collectively, OKS represents one of the best-supported enzymatic steps in CA biosynthesis, with strong evidence from biochemical characterization and heterologous reconstruction. Nevertheless, identification of the authentic insect-derived polyketide synthase will be required to fully establish the native biosynthetic pathway.

#### 3.2.2. Glycosylation Mechanism

The biosynthesis of CA involves a distinctive C-glycosylation reaction, which differentiates it from the more prevalent O-glycosylation reactions found in plant secondary metabolism. This transformation is catalyzed by specialized C-glucosyltransferases (CGTs), which establish a stable carbon–carbon bond between the sugar donor and the aromatic acceptor substrate [27,52].

A membrane-associated C-glucosyltransferase, commonly referred to as DcUGT2, has been identified as the key enzyme responsible for this transformation. Unlike most soluble UDP-dependent glycosyltransferases, DcUGT2 contains a C-terminal transmembrane region and is associated with the endoplasmic reticulum. Removal of this membrane-associated region significantly reduces catalytic activity, indicating that proper membrane localization is important for enzyme function [37]. DcUGT2 utilizes UDP-glucose as the sugar donor and directly catalyzes C-glycosylation of flavokermesic acid and kermesic acid to generate glucosylated anthraquinone intermediates, ultimately leading to CA formation [52]. Among the tailoring enzymes currently associated with CA biosynthesis, DcUGT2 represents the strongest experimentally supported native enzyme (Table 1). It was identified through transcriptomic analysis followed by biochemical characterization, and its catalytic activity has been confirmed through enzyme assays and heterologous expression systems [52]. In contrast, other reported CGTs from plants and microorganisms, including CGTs from buckwheat, mango, and gentian, have mainly been characterized using flavonoid substrates and serve primarily as engineering references for improving soluble C-glycosyltransferase platforms [35,54]. Although microbial CGT structures have provided important insights into the molecular basis of C–C bond formation [55], their direct applicability to anthraquinone C-glycosylation remains limited.

Mechanistically, C-glycosylation is more challenging than O-glycosylation because it requires precise positioning of the aromatic carbon nucleophile and formation of a chemically stable carbon–carbon bond. This unique catalytic requirement contributes to the stability of CA against hydrolysis, heat, and enzymatic degradation [52,55]. Therefore, improving the expression efficiency and substrate specificity of CGTs represents a major challenge for future microbial production of CA and related C-glycosylated natural products.

#### 3.2.3. Tailoring Enzymes

Following polyketide chain assembly, the formation of the anthraquinone core requires a series of tailoring reactions, including regioselective cyclization, aromatization, oxidation, and hydroxylation. These transformations convert the flexible linear octaketide intermediate into a structurally defined anthraquinone scaffold and determine the chemical framework required for subsequent glycosylation [26,28].

In heterologous reconstruction systems, cyclization specificity has been successfully controlled by the bacterial enzymes ZhuI and ZhuJ derived from *Streptomyces* sp. R1128. ZhuI functions as a cyclase that directs regioselective folding and ring closure of the linear octaketide intermediate, whereas ZhuJ contributes to subsequent aromatization and stabilization of the anthraquinone scaffold [26,43,56]. The combined activity of ZhuI and ZhuJ enabled efficient formation of flavokermesic acid-related intermediates in engineered hosts, demonstrating their functional compatibility with heterologous CA pathway reconstruction. However, these enzymes should be regarded as surrogate catalysts rather than confirmed native enzymes of *D. coccus*. The endogenous insect cyclases responsible for controlling octaketide folding and aromatization have not yet been experimentally identified.

Oxidative tailoring reactions represent another critical stage in anthraquinone maturation. In the reconstructed yeast pathway, AptC from *A. nidulans* was identified as an anthrone monooxygenase responsible for converting flavokermesic acid anthrone into flavokermesic acid. Through comparative screening of candidate oxidases and pathway reconstruction, AptC was experimentally demonstrated to promote this oxidative conversion and increase flux toward CA production [28,29]. Nevertheless, AptC represents a heterologous enzyme selected for pathway completion rather than the native oxidase operating in *D. coccus*.

Further hydroxylation is required for conversion of flavokermesic acid to kermesic acid, which provides the aglycone substrate for the final C-glycosylation step. Recent pathway reconstruction studies identified Cat5, an endogenous cytochrome P450 hydroxylase from *S. cerevisiae*, as an enzyme capable of performing this reaction in yeast-based production systems [29,43]. This finding highlights the functional flexibility of oxidative tailoring reactions; however, the native insect hydroxylase responsible for kermesic acid formation remains unresolved. Diverse cytochrome P450 hydroxylases from fungi and plants can be repurposed for anthraquinone modification [35].

Collectively, current knowledge indicates that tailoring reactions involved in CA biosynthesis have been established mainly through heterologous pathway engineering rather than complete characterization of the native insect pathway. While ZhuI, ZhuJ, AptC, and Cat5 have enabled successful reconstruction of anthraquinone maturation (Table 1), their roles should be interpreted according to their evidence levels: surrogate enzymes for scaffold formation, heterologous enzymes for oxidative conversion, and host-derived enzymes for pathway completion. Identification and biochemical characterization of the corresponding *D. coccus*-derived tailoring enzymes remain essential for fully elucidating the natural biosynthetic route.

#### 3.2.4. Current Experimental Evidence and Remaining Uncertainties

Although the biosynthetic pathway of CA has been reconstructed in several heterologous hosts, the level of experimental support for individual enzymatic steps varies considerably. Current understanding of the pathway has been established through a combination of biochemical characterization, heterologous pathway reconstruction, functional replacement, and comparative analysis. Therefore, distinguishing experimentally validated enzymes from inferred pathway assignments is essential for accurately evaluating the current state of CA biosynthesis. Among the currently identified enzymes, DcUGT2 represents the most strongly supported native enzyme associated with CA biosynthesis. DcUGT2 was identified from *D. coccus* through transcriptomic analysis and subsequently validated by biochemical assays, demonstrating direct C-glycosylation activity toward flavokermesic acid and kermesic acid. These results provide direct evidence linking DcUGT2 to the formation of CA and establish it as the best-characterized native tailoring enzyme reported to date [26].

In contrast, several enzymes involved in anthraquinone scaffold formation have been validated mainly through heterologous reconstruction rather than direct characterization from *D. coccus*. The plant-derived AaOKS and bacterial enzymes ZhuI and ZhuJ successfully enabled the formation of anthraquinone intermediates in engineered microbial hosts, demonstrating their catalytic compatibility with CA pathway reconstruction. However, these enzymes should be considered functional substitutes rather than confirmed native components of the insect pathway because their physiological counterparts in *D. coccus* remain unidentified [28]. Recent metabolic engineering studies further resolved several oxidative tailoring steps required for CA production. AptC from *A. nidulans* was identified as an anthrone monooxygenase responsible for oxidation of flavokermesic acid anthrone, whereas Cat5 from *S.cerevisiae* was shown to catalyze hydroxylation of flavokermesic acid during pathway reconstruction in yeast. Although these enzymes have been experimentally validated in heterologous systems, they represent pathway-enabling enzymes rather than confirmed native insect catalysts [29].

Overall, the currently accepted CA biosynthetic pathway should be regarded as a reconstructed enzymatic framework rather than a completely resolved native pathway. The identities of endogenous insect enzymes responsible for several key reactions, particularly cyclization, oxidation, and hydroxylation, remain uncertain. Future studies integrating insect genome mining, transcriptomic profiling, protein characterization, and in vitro enzyme reconstruction will be required to identify native pathway components and establish a complete mechanistic understanding of CA biosynthesis. From a synthetic biology perspective, the existing enzyme repertoire provides a valuable foundation for microbial production of CA. Nevertheless, replacing surrogate enzymes with native insect-derived catalysts and improving the catalytic performance of bottleneck enzymes, particularly C-glycosyltransferases and oxidative tailoring enzymes, remain major challenges for future pathway optimization [26,28,29].

## 4. Microbial Production of Carminic Acid

While the biosynthetic pathway of CA has been increasingly elucidated, its efficient implementation in microbial systems remains challenging due to limitations in precursor supply, enzyme compatibility, and cellular context [1,28,29,43]. Four mainstream chassis (*E. coli*, *S. cerevisiae*, *Y. lipolytica*, and *A. nidulans*) have been established for CA de novo synthesis, each with unique metabolic advantages and intrinsic bottlenecks [28,29,43,44,57]. Universal microbial cell factory engineering frameworks cover precursor enhancement, dynamic flux control, subcellular compartmentalization and protein engineering [31,32].

### 4.1. Chassis Selection and Pathway Establishment

The selection of an appropriate chassis plays a critical role in determining pathway feasibility and productivity, as different hosts vary significantly in their metabolic capacity, enzyme compatibility, and intracellular organization (Table 2). In general, these systems represent distinct strategies for pathway implementation, ranging from rapid prototyping in prokaryotes to more native-like reconstruction in eukaryotic hosts [28,29,43].

#### 4.1.1. *E. coli* as a Heterologous Host

*E. coli* has been extensively utilized as a model chassis for heterologous biosynthesis owing to its rapid growth, well-characterized genetics, and versatile metabolic engineering toolkit [58]. These features make it particularly suitable for pathway prototyping and functional validation of complex natural product biosynthetic routes. Complete modular polyketide synthesis systems have been realized in engineered *E. coli* strains [23]. The feasibility of de novo CA production in *E. coli* has been demonstrated through reconstruction of the core biosynthetic pathway from central carbon metabolism. In this system, octaketide formation can be achieved using type II PKS with luminescent bacillus (AntDEFBG), followed by the introduction of cyclases such as ZhuI and ZhuJ to direct regioselective cyclization toward flavokermesic acid intermediates [43].

Notably, the reconstructed pathway in *E. coli* deviates from the native biosynthetic route in several key aspects. Due to the limited compatibility of insect-derived enzymes with the prokaryotic cellular environment, particularly membrane-associated proteins, native enzymes are often replaced with functionally analogous counterparts. For example, surrogate enzymes such as the anthraquinone hydroxylase DerF from *Streptomyces peucetius* and plant-derived C-glycosyltransferase GtCGT from Gentiana triflora have been employed to complete downstream transformations. This results in a “rewired” biosynthetic pathway shaped by host constraints [43]. A major bottleneck arises from the inability to functionally express the native C-glycosyltransferase DcUGT2 in *E. coli*, even after extensive engineering efforts targeting its signal peptide and transmembrane domains. Consequently, production levels remain low, reflecting limitations in enzyme compatibility, precursor supply, and cellular context for complex tailoring reactions [43]. Comprehensive reviews summarize strengths and defects of *E. coli* as a polyketide chassis [58,59].

In *E. coli*, the titer improvement trajectory reveals a clear hierarchy of bottlenecks. Initial pathway reconstruction yielded only 22.2 μg/L in shake flasks. Overexpression of acetyl-CoA carboxylase (ACC) to enhance malonyl-CoA supply resulted in the largest single-gene improvement (3.4-fold, to 74.9 μg/L), establishing precursor availability as the primary limiting factor. Molecular chaperone co-expression (DnaK-DnaJ-GrpE) provided a more modest 2.2-fold improvement, partially alleviating protein folding issues of heterologous enzymes. Notably, fed-batch fermentation achieved the most substantial increase (~8.4-fold relative to shake-flask ACC-overexpressing strain), reaching 0.63 mg/L. This pattern indicates that while pathway enzymes function suboptimally in *E. coli*, the primary constraint at scale is not enzyme activity per se but rather the capacity to sustain high metabolic flux during prolonged cultivation [43]. Collectively, *E. coli* serves as a powerful platform for rapid pathway assembly and mechanistic validation, but its intrinsic limitations necessitate pathway rewiring and constrain efficient production.

#### 4.1.2. Yeast-Based Systems: *S. cerevisiae* and *Y. lipolytica*

In contrast to bacterial systems, yeast hosts provide a eukaryotic cellular environment that is more compatible with membrane-associated enzymes and post-translational modifications. This makes them particularly suitable for reconstructing biosynthetic pathways involving complex tailoring reactions such as C-glycosylation [29,52]. Multiple flavonoid and polyketide pathways have been successfully reconstructed in yeast, including naringenin and rubrofusarin biosynthesis [60,61,62].

Reconstitution of the CA pathway in *S. cerevisiae* has demonstrated improved compatibility with key enzymes derived from the native producer. The team from Jiangnan University excavated the 4′-phosphopantetheinyl transferase (PPTase) NpgA and monooxygenase AptC, which are involved in the synthesis of CA, and identified the endogenous hydroxylase Cat5 of *S. cerevisiae* as the key enzyme for the hydroxylation of cochineal, and reconstituted the complete cochinelicate biosynthesis pathway. Subsequently, *S. cerevisiae* was engineered by regulating enzyme expression, enhancing precursor supply, and modifying the fatty acid β-oxidation pathway [29]. In particular, the membrane-bound C-glycosyltransferase DcUGT2 can be functionally expressed in yeast, where its proper localization to the endoplasmic reticulum enables efficient C-glycosylation of anthraquinone intermediates. This allows a pathway configuration that more closely resembles the native biosynthetic route compared to bacterial systems [29,52].

In *S. cerevisiae*, the stepwise engineering strategy yielded more incremental improvements. The base strain CA10 produced 2.66 mg/L in shake flasks. Enhancing acetyl-CoA supply through β-oxidation engineering (FOX1 overexpression) and enzyme fusion (OKS-NpgA) each provided ~50% improvements, reaching ~4.07 mg/L. Glycosylation optimization (EXG1 knockout) had a smaller effect (~14%), suggesting that glycosylation is not the major bottleneck in yeast. Fed-batch fermentation further increased production to 7.58 mg/L (~1.9-fold), a smaller relative gain than in *E. coli* or *Y. lipolytica*, possibly because yeast’s tighter metabolic regulation limits the benefits of extended cultivation [29]. However, the implementation of upstream steps in *S. cerevisiae* remains challenging. Polyketide synthesis and cyclization efficiency can be limited by suboptimal coordination between enzymes, while oxidative tailoring steps may depend on auxiliary redox partners or host-derived enzymes. These factors often lead to imbalanced pathway flux and restrict overall production efficiency [29,44].

In addition to *S. cerevisiae*, the oleaginous yeast *Y. lipolytica* has emerged as a promising alternative host for polyketide production. Compared to S. cerevisiae, *Y. lipolytica* exhibits a metabolism more naturally geared toward acetyl-CoA and malonyl-CoA generation, which are key precursors for polyketide biosynthesis. This intrinsic metabolic advantage makes it particularly attractive for enhancing flux toward anthraquinone intermediates [44,57,63]. Extensive reviews detail lipid metabolism engineering in this chassis [64]. *Y. lipolytica* represents the most successful chassis to date, achieving 9.6 mg/L in fed-batch fermentation, setting a record for the highest yield recorded so far [65]. It provides an important reference for the efficient microbial synthesis of polyketones [57,66].

The improvement cascade is particularly instructive: starting from 0.7 mg/L with the basic pathway, ACC1 mutant screening combined with the NCM pathway increased titer to 1.2 mg/L (71% improvement), confirming malonyl-CoA as a key bottleneck. Lipid metabolism engineering (PEX10 + ACL1/2) further boosted production to 1.5 mg/L (25% improvement), leveraging *Y. lipolytica*’s inherent strength in acetyl-CoA generation. Peroxisomal compartmentalization added another 27% improvement (to 1.9 mg/L), demonstrating the value of spatial organization. The most dramatic improvement came from fed-batch fermentation with antioxidant supplementation (ascorbic acid), which increased titer ~5-fold to 9.6 mg/L [65]. This large fermentation effect suggests that the intrinsic pathway capacity of *Y. lipolytica* is significantly higher than shake-flask results imply, and that product/intermediate stability may be an underappreciated constraint. Nevertheless, while *Y. lipolytica* offers improved precursor supply, challenges related to pathway balancing, enzyme expression, and product toxicity remain to be addressed. As such, both yeast systems highlight a trade-off between metabolic capacity and pathway complexity, underscoring the need for integrated engineering strategies.

#### 4.1.3. Filamentous Fungi: *A. nidulans*

Filamentous fungi such as *A. nidulans* represent an alternative class of hosts that are naturally equipped for polyketide biosynthesis. Unlike bacterial and yeast systems, these organisms possess endogenous polyketide synthase machinery and regulatory networks tailored for the production of aromatic secondary metabolites [28,67]. The use of *A. nidulans* enables a more native-like biosynthetic context for anthraquinone production, particularly with respect to polyketide chain assembly and cyclization. The presence of compatible intracellular environments and accessory enzymes facilitates the formation of complex aromatic scaffolds with reduced need for extensive pathway rewiring. The most prominent advantage is that it contains a variety of oxidases endogenously [67], which can spontaneously complete the oxidation step of flavokermesic acid anthrone to form flavokermesic acid, without the need for additional heterologous expression of oxidase. In addition, 4′-phosphopantetheinyl transferase (PPTase) is also contained as a cofactor, which is involved in the activation process of OKS and is crucial for synthesis efficiency [28].

Systems biology approaches have further advanced our understanding of fungal polyketide production in *A. nidulans*. For example, combined transcriptomic, fluxomic, and physiological analyses have enabled the construction of interaction models describing the competition between biomass formation and polyketide production for acetyl-CoA, providing valuable insights into metabolic regulation that can inform future engineering strategies [67].

However, despite these advantages, filamentous fungi present their own challenges, including slower growth rates, more complex genetic manipulation, and less well-characterized metabolic networks compared to model hosts. These factors can limit their scalability and ease of engineering. Overall, *A. nidulans* provides a complementary platform that bridges the gap between fully engineered microbial systems and native producers, offering valuable insights into pathway fidelity and enzyme compatibility. From a systematic comparison, *Y. lipolytica* stands out as the most promising chassis for industrial titer improvement. Its inherently active acetyl-CoA and malonyl-CoA metabolism, driven by robust lipid biosynthesis pathways, aligns well with the precursor demand of polyketide biosynthesis. The combined effects of ACC1 mutation, NCM pathway implementation, and peroxisomal compartmentalization have delivered stepwise titer gains, outperforming S. cerevisiae in fed-batch fermentation [67]. In contrast, *S. cerevisiae* benefits from more mature genetic tools and better-characterized metabolism, making it more suitable for mechanistic studies and pathway debugging. *E. coli* remains the preferred platform for initial pathway assembly and rapid enzyme screening, despite its low final production level [47]. Overall, despite successful pathway reconstruction in multiple hosts, production levels remain relatively low and highly context-dependent, underscoring the need for further optimization at both metabolic and enzymatic levels.

### 4.2. Precursor Supply Engineering

The efficient microbial biosynthesis of CA is fundamentally constrained by the availability of malonyl-CoA, the essential extender unit for iterative polyketide chain elongation. Across diverse hosts, intracellular malonyl-CoA pools are inherently limited due to their tight regulation and preferential allocation to primary metabolism, particularly fatty acid biosynthesis [44,68,69]. This establishes precursor supply as a primary bottleneck, positioning it as a foundational target for metabolic engineering. Efforts to enhance malonyl-CoA availability have evolved along three distinct yet interconnected strategies: reinforcement of native pathways, implementation of orthogonal biosynthetic routes, and development of fully decoupled synthetic pathways.

#### 4.2.1. Reinforcing Native Precursor Pathways

Initial strategies have focused on amplifying the canonical acetyl-CoA-dependent route, in which malonyl-CoA is generated via ATP-dependent carboxylation catalyzed by acetyl-CoA carboxylase (ACC). Enhancing this axis typically involves increasing acetyl-CoA supply through central metabolic rewiring and elevating ACC activity via overexpression or deregulation [44,68,70].

Such interventions can effectively expand the malonyl-CoA pool and improve polyketide production. This strategy has been successfully demonstrated across diverse fungal hosts: for example, overexpression of acetyl-CoA carboxylase in *A. terreus* increased malonyl-CoA levels by 240% and boosted lovastatin production by 40%, providing a precedent for ACC-based precursor engineering in filamentous fungi [71]. Similarly, in *A. oryzae*, strengthening malonyl-CoA supply through ACC enhancement and sterol pathway disruption has been shown to improve the yields of plant polyketides such as curcumin and atrochrysone carboxylic acid-related compounds, underscoring the broad applicability of this approach across fungal systems [72,73].

However, the inherent limitations of this pathway—including low catalytic efficiency, high ATP demand, and multilayered regulatory constraints—restrict its capacity to sustain high flux toward secondary metabolism. Moreover, the central role of acetyl-CoA in essential cellular processes imposes a fundamental trade-off between precursor supply and cellular fitness [44]. This highlights that native pathway reinforcement, while necessary, is insufficient to overcome systemic flux limitations.

#### 4.2.2. Orthogonal Pathways for Malonyl-CoA Biosynthesis

To alleviate the constraints imposed by native metabolism, orthogonal strategies have been developed to partially bypass endogenous regulation. Among these, malonate assimilation pathways enable the direct conversion of exogenously supplied malonate into malonyl-CoA, providing an alternative and tunable precursor source [68]. This approach decouples malonyl-CoA formation from acetyl-CoA availability and allows external control over intracellular precursor levels. As a result, significant increases in malonyl-CoA pools and improved incorporation into polyketide products have been achieved, particularly in bacterial systems where native precursor supply is limited [68]. Nevertheless, these strategies remain dependent on substrate supplementation and do not fully eliminate interactions with host metabolic networks, thereby limiting their scalability and robustness.

#### 4.2.3. Decoupled Synthetic Pathways: The NCM Paradigm

Recent advances have shifted toward the design of synthetic pathways that fundamentally decouple malonyl-CoA biosynthesis from central metabolism. The non-carboxylative malonyl-CoA (NCM) pathway exemplifies this paradigm by enabling the direct conversion of pyruvate-derived intermediates into malonyl-CoA without proceeding through acetyl-CoA [57]. By bypassing the canonical decarboxylation–carboxylation cycle, the NCM pathway eliminates ATP consumption and carbon loss, while exhibiting substantially improved catalytic efficiency compared to the native route. Importantly, it circumvents multiple layers of endogenous regulation associated with the pyruvate dehydrogenase and ACC nodes, thereby reducing metabolic cross-talk and regulatory interference [57].

From an engineering perspective, this strategy transforms malonyl-CoA biosynthesis from a tightly constrained metabolic node into a modular and orthogonal function, enabling high precursor flux without perturbing essential cellular processes. In yeast, multiple strategies including ACC mutant screening, lipid pathway knockout and NCM heterologous expression are combined to maximize malonyl-CoA flux [74,75]. The successful implementation of such decoupled pathways in microbial hosts further demonstrates their capacity to substantially enhance the production of malonyl-CoA-derived compounds. This suggests that metabolic decoupling, rather than pathway amplification, represents a more effective route to overcoming intrinsic limitations in precursor supply, particularly for complex polyketides such as CA.

### 4.3. Metabolic Flux Reprogramming and Dynamic Regulation

The efficient biosynthesis of CA is heavily influenced by metabolic flux redistribution, particularly the allocation of malonyl-CoA, a key precursor in the polyketide biosynthesis pathway. Traditional metabolic engineering approaches often focus on increasing precursor availability, but even with enhanced malonyl-CoA pools, competition for this critical metabolite with fatty acid biosynthesis and other pathways remains a major bottleneck. Effective flux reprogramming therefore requires not only increasing precursor supply but also dynamically redirecting metabolic flux to the desired pathway [21,76,77].

Recent advances in smart biosensing technologies have further expanded the toolbox for dynamic metabolic engineering. Biosensors not only enable precise genetic regulation but also provide real-time monitoring and external interfacing capabilities with diverse signal modalities, including electrical and optical systems. By incorporating dynamic control mechanisms, synthetic pathways become more robust to environmental fluctuations during scale-up and more precisely regulated in therapeutic contexts, which is critical for advancing the reliability and applicability of engineered metabolic systems [21].

#### 4.3.1. Dynamic Control and Hierarchical Regulation

Recent advances in dynamic metabolic control have enabled more effective management of intracellular flux, particularly in response to metabolic state changes. Dynamic regulation allows cells to adjust pathway expression levels based on internal signals, such as metabolite concentrations, rather than relying on static overexpression of pathway enzymes. One key advance in this area has been the development of genetically encoded biosensors that respond to metabolites like malonyl-CoA, which can be used to dynamically control the expression of heterologous pathways [19,21,77,78,79]. Early work on malonyl-CoA sensing established the foundation for subsequent dynamic regulation strategies. For instance, the design and kinetic analysis of a hybrid promoter–regulator system for malonyl-CoA sensing in *E. coli* provided early insights into how biosensor-regulator circuits can be engineered to respond to intracellular metabolite levels, paving the way for more sophisticated dynamic control systems [79].

For example, the FapR-based malonyl-CoA sensor has been employed in *S. cerevisiae* to regulate the expression of pathways involved in the production of 3-hydroxypropionic acid (3-HP). This approach uses a dual-layer control system where the upper level modulates the expression of enzymes involved in fatty acid biosynthesis, which competes for malonyl-CoA, while the lower level uses the malonyl-CoA sensor to activate the heterologous 3-HP biosynthetic pathway. The result is a significant increase in 3-HP production, demonstrating the effectiveness of hierarchical dynamic control [76,80]. This control system has also been demonstrated in *E. coli*, where a similar sensor-regulator circuit was constructed using FapR to tune malonyl-CoA levels dynamically [79]. By adjusting the production of enzymes that utilize malonyl-CoA, these engineered systems allow the cells to more efficiently redistribute flux toward the target product, enhancing overall production yields [78,79].

Beyond single-metabolite sensors, multi-layered dynamic regulation networks have been developed to achieve more sophisticated flux control. For example, a growth-coupled multi-layered dynamic regulation network (DRN) balancing the malonyl-CoA node was engineered for enhanced (*2S*)-naringenin biosynthesis in *E. coli*. This NCOMB DRN system concurrently regulates multiple genes in both malonyl-CoA catabolic and anabolic pathways, using a biosensor-based directed evolution strategy to optimize synergies between different dynamic regulation layers, resulting in substantially improved production titers [18]. Additionally, quorum-sensing-based dynamic regulatory circuits have been engineered in *S. cerevisiae* using N-acyl-homoserine lactone systems, providing another layer of autonomous population-level control that can coordinate pathway expression across cell populations without relying on external inducers [19]. Furthermore, synthetic metabolic oscillators based on malonyl-CoA have been constructed in *Komagataella phaffii*, demonstrating that metabolic state can be engineered to generate rhythmic behaviors, which opens new possibilities for temporal regulation of biosynthetic pathways [81]. Beyond single-metabolite sensors, machine learning-aided flux balancing approaches have emerged as powerful tools for pathway optimization. For instance, a bottlenecking-debottlenecking strategy combined with machine learning models has been applied to balance the naringenin biosynthetic pathway, enabling parallel evolution of all pathway enzymes along predictable trajectories and achieving significantly improved titers [82,83]. Similarly, systems-level metabolic engineering approaches that integrate pathway balancing, enzyme screening, and promoter tuning have demonstrated substantial titer improvements for complex natural products, providing a generalizable framework for flux optimization [84].

Overall, machine learning and deep learning have become increasingly integrated into synthetic biology and metabolic engineering. These computational approaches enable predictive modeling of pathway behavior, optimal experimental design, and data-driven strain engineering, complementing traditional mechanistic models and reducing the need for exhaustive experimental screening [85]. Active learning approaches, in particular, have shown promise in intelligently guiding experiments to arrive at optimal phenotypes with minimal measured datasets, as demonstrated in case studies of valine yield improvement and neurosporene productivity enhancement in *E. coli* [83].

#### 4.3.2. Pathway Repression and Competition for Malonyl-CoA

One of the most critical challenges in flux reprogramming is overcoming the competition for malonyl-CoA between the target biosynthetic pathway and fatty acid biosynthesis. Fatty acids, a major sink for malonyl-CoA, often limit the efficiency of polyketide production. As malonyl-CoA is the rate-limiting precursor in both fatty acid and polyketide biosynthesis, optimizing the allocation of this metabolite is essential for improving yields [44,77]. To address this, strategies have been developed to repress fatty acid biosynthesis in favor of polyketide production. One effective method is the downregulation of key enzymes in the fatty acid biosynthetic pathway, such as acetyl-CoA carboxylase (ACC), which catalyzes the conversion of acetyl-CoA to malonyl-CoA. By using CRISPR interference (CRISPRi) or other gene silencing methods, the flux toward fatty acid synthesis can be reduced, thereby increasing the availability of malonyl-CoA for polyketide production [77,86].

Additionally, the use of feedback loops has been explored as a way to control flux dynamically. By integrating the malonyl-CoA biosensor into the feedback loop, the production of fatty acids can be suppressed when malonyl-CoA levels exceed a certain threshold. This prevents the diversion of malonyl-CoA into non-target pathways, thus improving the efficiency of polyketide biosynthesis [76,78].

#### 4.3.3. Flux Redistribution Through Engineered Pathways

Beyond controlling competition for malonyl-CoA, flux redistribution can be achieved through engineered pathway modifications. For example, in the case of *E. coli*, the introduction of a malonate assimilation pathway provides an alternative source of malonyl-CoA, which can be utilized by the polyketide biosynthesis pathway, thus alleviating the burden on the native acetyl-CoA–dependent malonyl-CoA synthesis pathway. This approach allows for decoupling of malonyl-CoA biosynthesis from central metabolism, making it possible to increase the flux toward polyketide production without overloading the host’s central metabolic network [68].

Another promising strategy is the use of two-stage fermentation processes, which decouple biomass accumulation from product formation [79]. In the first stage, the system focuses on cell growth, and in the second stage, metabolic flux is redirected toward product synthesis. This strategy has been successfully applied in several microbial production systems, demonstrating how dynamic shifts in metabolic flux can be used to maximize product yields [77].

Beyond biosensor-based dynamic regulation, additional advanced engineering strategies can be applied to further optimize CA biosynthetic pathways. CRISPR interference (CRISPRi) enables tunable, multiplexed repression of competing pathways such as fatty acid biosynthesis without permanent gene knockout, allowing precise flux redirection at the malonyl-CoA node [87]. Adaptive laboratory evolution (ALE) can be used to improve host tolerance to anthraquinone toxicity and select for strains with enhanced precursor supply. Genome-scale metabolic models (GEMs) can systematically predict non-intuitive engineering targets for titer improvement, guiding rational strain engineering [31]. Cell-free biosynthesis systems represent another emerging approach for rapid pathway prototyping and enzyme characterization, decoupling biosynthetic reactions from cellular growth constraints.

### 4.4. Enzyme Compatibility and Pathway Optimization

Beyond precursor availability, the successful reconstruction of the CA pathway critically depends on the functional expression and compatibility of heterologous enzymes. Differences in protein folding, localization, and cofactor requirements across hosts frequently result in incomplete or inefficient pathway operation [28,29,43]. Activation of the polyketide synthase represents a primary constraint. Type III PKSs such as octaketide synthase require post-translational modification to become catalytically competent, indicating that pathway reconstruction must account for auxiliary enzymes required for protein maturation. This highlights that enzyme functionality is often context-dependent and cannot be assumed from sequence alone [28].

A major challenge arises from the expression of membrane-associated enzymes, particularly the C-glycosyltransferase responsible for stabilizing the anthraquinone scaffold. While such enzymes can be functionally expressed in eukaryotic hosts, they are frequently incompatible with prokaryotic systems. This necessitates pathway redesign through enzyme substitution, where soluble homologs are identified and adapted to replace native enzymes [43,52]. Rational enzyme engineering further enhances pathway efficiency. Structure-guided mutagenesis and substrate docking analyses enable targeted improvements in catalytic activity and substrate specificity, particularly for glycosyltransferases and hydroxylases. In addition, repurposing enzymes from unrelated biosynthetic pathways provides an effective strategy to fill missing catalytic steps [27,51].

Furthermore, recent advances in protein engineering have significantly expanded the toolkit for optimizing industrial biocatalysis, with successful applications across diverse high-value compounds including CA, α-farnesene, vitamin B12, and patchoulol, among others. These advances underscore the transformative potential of protein engineering for enhancing microbial production of valuable substances [87]. More broadly, rational protein design has emerged as a powerful approach for tailoring enzyme properties. Strategies including multiple sequence alignment, steric hindrance-based design, interaction network remodeling, and dynamics modification have been successfully applied to enhance enzyme activity and enantioselectivity, providing a general framework for enzyme optimization [88].

Complementing these approaches, artificial intelligence and deep learning technologies have revolutionized enzyme prediction and rational design. Advances in protein structure prediction, particularly AlphaFold-driven structural modeling, have generated millions of protein structure models and provided unprecedented structural information for understanding enzyme function and guiding engineering efforts [89,90]. Structure-guided rational design, supported by AlphaFold-driven protein structure prediction, has been widely applied to engineer type III PKSs and glycosyltransferases for improved activity, substrate specificity, and regioselectivity [90,91]. For CA pathway enzymes, AlphaFold models can be used to resolve the catalytic pocket architecture of OKS and DcUGT2, guiding targeted mutagenesis to reduce shunt product formation and enhance C-glycosylation efficiency. Multiple review articles systematically summarize AI-assisted enzyme design industrial application prospects [91,92]. So far, no AI-designed enzyme specifically for CA biosynthesis has been experimentally validated to date. The application of AI-assisted enzyme engineering in this field remains at the prospective stage. Nevertheless, related advances in other polyketide and glycosyltransferase systems provide strong methodological precedents. Furthermore, machine learning-based protein fitness prediction frameworks have demonstrated the ability to accurately predict sequence-function relationships, enabling efficient identification of high-fitness enzyme variants without exhaustive experimental screening [93]. Looking forward, AI-assisted enzyme engineering represents one of the most promising avenues for breaking the intrinsic efficiency ceiling of the CA biosynthetic pathway, particularly for improving the catalytic fidelity of OKS and the specific activity of membrane-bound CGTs [38]. Machine learning has also emerged as a transformative force in accelerating biocatalysis discovery. Emerging computational tools promise to revolutionize protein engineering for biocatalytic applications, with predictive algorithms helping scientists navigate the complexity of functional protein sequence space and reducing the development timelines previously needed to optimize enzymes [92]. Machine learning-based protein fitness prediction frameworks further enable high-throughput screening of enzyme variants without exhaustive experimental testing, accelerating directed evolution campaigns [93].

At the gene expression level, untranslated region (UTR) engineering strategies offer additional avenues for fine-tuning enzyme expression levels. UTR engineering enables precise control over gene expression levels, allowing both overexpression and fine-tuning of pathway enzymes, and can also be integrated with dynamic regulation strategies to achieve more sophisticated temporal and metabolic state-dependent control of pathway flux [80]. These findings collectively indicate that pathway optimization is not merely a matter of assembling enzymes, but of ensuring their functional integration within the host cellular environment.

### 4.5. Cellular Organization and Bioprocess Integration

The efficiency of CA biosynthesis is further shaped by the cellular context in which the pathway operates, including both subcellular organization and process-level conditions. These factors collectively determine enzyme accessibility, metabolite channeling, and overall pathway performance [66,94]. Host selection plays a decisive role in enabling pathway functionality. Eukaryotic systems provide a favorable environment for membrane-associated and compartmentalized reactions, allowing pathway configurations that more closely resemble native biosynthesis. In contrast, prokaryotic hosts often require fully heterologous and restructured pathways due to limitations in protein localization and processing. This underscores the importance of host–pathway compatibility as a design principle.

Spatial organization through subcellular compartmentalization provides an additional layer of control. Targeting biosynthetic pathways to organelles such as peroxisomes enables the sequestration of intermediates, reduces interference from competing pathways, and increases local concentrations of enzymes and substrates. Currently, research has optimized the peroxisome capacity of *S. cerevisiae* through machine learning, further improving multi-enzyme pathway efficiency and significantly increasing the yield of geraniol [95]. Moreover, compartmentalization can facilitate direct access to localized precursor pools, such as acetyl-CoA derived from β-oxidation. These advantages highlight spatial engineering as a powerful strategy to enhance metabolic efficiency [66,94]. Beyond organelle targeting, substrate channeling through enzyme complexes and spatial organization represents another important mechanism for enhancing pathway efficiency. By physically associating consecutive enzymes in a pathway, substrate channeling can increase local metabolite concentrations, reduce intermediate loss, and minimize competition with side reactions, a principle that has been widely exploited in biotechnological applications [96]. From a biophysical perspective, cellular compartmentalization also functions as a physical regulatory mechanism, isolating biochemical processes using membrane barriers and influencing the dynamics and strength of cellular signaling through controlled transport rates across compartments [97]. Furthermore, the intersection between metabolism and translation through a subcellular lens reveals that metabolic pathways and protein synthesis are intricately organized across different compartments, adding another dimension of complexity and regulatory potential to biosynthetic engineering [98].

At the process level, fermentation strategies are essential for translating engineered pathways into productive systems. High-cell-density cultivation, controlled nutrient feeding, and maintenance of optimal environmental parameters collectively support sustained production. In particular, fed-batch processes enable the decoupling of growth and production phases, thereby improving overall yields [29]. Taken together, these results demonstrate that cellular organization and bioprocess conditions function synergistically with genetic engineering, forming an integrated framework for optimizing CA production.

Quantitative analysis showed that the increase in carminic acid yield exhibited a hierarchical characteristic: process optimization > precursor engineering > pathway optimization. Optimizing the fermentation process contributed the largest yield increase (5–8 times), but this was mainly achieved by increasing biomass and extending production time, rather than improving the efficiency per cell production. Precursor supply engineering (1.7–3.4 times) is the second largest contributing factor, reflecting that malonyl-CoA availability is a common bottleneck for polyketo biosynthesis. In contrast, engineering modifications for individual enzymes contribute relatively little to overall yield (usually < twice as much), because rate-limiting steps in multi-enzyme pathways dynamically shift, and single-enzyme enhancement is often diluted by bottlenecks in other steps (Table 3).

### 4.6. Why Has Microbial Carminic Acid Production Plateaued Below 10 mg L^−1^ Despite Extensive Metabolic Engineering?

Based on the quantitative analysis of engineering contributions (Table 3), the primary bottleneck limiting CA production is the intrinsic catalytic inefficiency of the pathway and oxidative instability of intermediates, followed by malonyl-CoA precursor supply. Single-enzyme optimization yields the smallest marginal benefit, as rate-limiting steps dynamically shift along the long multi-enzyme cascade.

Despite remarkable advances in pathway reconstruction, precursor engineering, enzyme optimization, and fermentation process development, the reported microbial production of CA has remained at the milligram-per-liter level, with the reported microbial titers remaining below 10 mg L^−1^ in most engineered systems [28,29,43,65]. This ceiling is strikingly lower than those achieved for many other microbial natural products, including flavonoids, terpenoids, and several aromatic polyketides. The limited productivity cannot be attributed to a single rate-limiting enzyme or insufficient precursor supply. Instead, it reflects the combined effects of intrinsic pathway inefficiency, distributed metabolic control, cross-kingdom enzyme incompatibility, rigid metabolic regulation, and poor stability of pathway intermediates. These constraints interact with each other across different biological scales, resulting in a highly resistant biosynthetic system in which improvement of one bottleneck frequently exposes another.

#### 4.6.1. Intrinsic Inefficiency of the Carminic Acid Biosynthetic Pathway

Unlike many microbial secondary metabolic pathways that have evolved to channel carbon efficiently toward a single end product, the reconstructed CA pathway is intrinsically inefficient from its very first catalytic step. Octaketide synthase (OKS), a type III polyketide synthase, produces a highly reactive linear octaketide intermediate that readily undergoes spontaneous or alternative cyclization [28,56]. Consequently, only a small proportion of the synthesized polyketide is converted into the desired flavokermesic acid precursor. Beyond spontaneous cyclization side reactions, the type III PKS system lacks the acyl carrier protein (ACP)-mediated substrate channeling present in type I/II PKS, leading to frequent premature chain release and shunt product formation [52]. This structural feature explains why OKS alone generates multiple off-pathway polyketide products even under optimal expression conditions [28]. Evidence from *A. nidulans* illustrates this limitation. Expression of OKS alone generates at least six distinct polyketide products, including SEK4, SEK4b, dehydro-SEK4, dehydro-SEK4b [28], mutactin, and flavokermesic acid, indicating that the majority of metabolic flux is dissipated into undesired side products. Although introduction of the bacterial cyclase ZhuI and aromatase ZhuJ increases productive cyclization by approximately fourfold, considerable carbon loss still occurs through competing reactions [28].

A second intrinsic limitation is the terminal C-glycosylation reaction. Unlike the more common O-glycosylation, C-glycosylation requires direct formation of a carbon–carbon bond and therefore imposes much stricter catalytic requirements [37,52]. Moreover, the native insect C-glycosyltransferase DcUGT2 is an endoplasmic-reticulum-associated membrane protein whose activity depends on the eukaryotic membrane environment [52]. Functional expression of DcUGT2 has not yet been achieved in *E. coli*, forcing bacterial systems to rely on surrogate plant-derived C-glycosyltransferases such as GtCGT, which exhibit lower catalytic efficiency and altered substrate specificity [43]. Consequently, both the first committed step and the terminal glycosylation reaction exhibit inherently low catalytic efficiency, imposing fundamental limits on pathway productivity. Future improvement therefore requires enhancing catalytic fidelity rather than simply increasing enzyme abundance. Structure-guided protein engineering, AI-assisted enzyme design, and directed evolution of both OKS and C-glycosyltransferases are expected to reduce carbon leakage and improve overall pathway efficiency.

#### 4.6.2. Flux Dilution Across a Long Multienzyme Pathway

In addition to the intrinsic inefficiency of individual enzymes, the overall architecture of the pathway also limits productivity. Biosynthesis of CA requires the coordinated action of at least eight enzymatic reactions, including PPTase-mediated activation, polyketide assembly, cyclization, aromatization, oxidation, hydroxylation, and C-glycosylation. According to metabolic control analysis (MCA), metabolic flux in long biosynthetic pathways is distributed among multiple enzymes rather than controlled by a single rate-limiting step [99]. As pathway length increases, the flux control coefficient of each individual enzyme decreases, meaning that improving one enzyme often results in only modest increases in overall pathway flux before control shifts to another downstream reaction.

This phenomenon has been consistently observed during CA pathway optimization, where engineering strategies such as precursor enhancement, molecular chaperone expression, enzyme fusion, compartmentalization, and glycosyltransferase optimization generally provide incremental rather than transformative improvements. The continuous migration of bottlenecks suggests that pathway optimization has entered a stage of diminishing returns. Consequently, future engineering should move beyond sequential optimization of individual enzymes toward systems-level balancing of the entire pathway through combinatorial regulation of enzyme expression, dynamic control of pathway flux, and coordinated optimization of enzyme stoichiometry and spatial organization.

#### 4.6.3. Cross-Kingdom Incompatibility Between Pathway Enzymes and Microbial Hosts

CA biosynthesis presents an additional challenge because the reconstructed pathway combines enzymes originating from multiple biological kingdoms. The current heterologous pathway incorporates a plant-derived type III PKS, bacterial cyclases, fungal or bacterial hydroxylases, and an insect membrane-bound C-glycosyltransferase within a single microbial host [28]. Such an artificial combination inevitably creates incompatibilities at multiple levels, including protein folding, post-translational modification, membrane localization, cofactor availability, and interactions with endogenous metabolic networks. The inability to functionally express DcUGT2 in bacterial hosts illustrates this incompatibility, necessitating replacement with non-native enzymes that only partially reproduce the catalytic function of the original pathway [43,52]. Although codon optimization, molecular chaperones, and enzyme substitution have improved heterologous expression, these approaches rarely restore native catalytic performance completely.

As a result, microbial production relies on a reconstructed hybrid pathway rather than faithful replication of the native biosynthetic machinery. Future progress will therefore depend not only on improving heterologous expression but also on increasing host–pathway compatibility through the discovery of more suitable orthologs, computational redesign of enzymes, and the development of eukaryotic chassis capable of supporting membrane-associated and compartment-dependent reactions.

#### 4.6.4. Rigid Metabolic Constraints on Precursor Allocation

Even if all pathway enzymes function optimally, precursor availability remains fundamentally constrained by host metabolism. Acetyl-CoA and malonyl-CoA occupy central positions in cellular carbon metabolism and simultaneously serve as essential precursors for fatty acid biosynthesis and membrane formation. Consequently, increasing malonyl-CoA availability inevitably competes with cell growth and biomass accumulation [44]. At the molecular level, acetyl-CoA carboxylase (ACC), the rate-limiting enzyme for malonyl-CoA synthesis, is subject to multi-layered regulation: transcriptional repression by fatty acid end products, post-translational inhibition via Snf1-mediated phosphorylation, and allosteric feedback by long-chain acyl-CoAs [48,71]. These regulatory mechanisms ensure that malonyl-CoA is preferentially allocated to essential fatty acid biosynthesis for membrane biogenesis, creating a fundamental trade-off between polyketide production and cell growth.

In addition to carbon precursor imbalance, reductive cofactor limitation represents an underrecognized metabolic bottleneck. The oxidative tailoring steps—including anthrone monooxygenation and P450-catalyzed hydroxylation—consume stoichiometric amounts of NADPH. In yeast, NADPH is primarily supplied by the pentose phosphate pathway, and high flux through polyketide biosynthesis can deplete the cellular NADPH pool, restricting the activity of oxidative tailoring enzymes and reducing pathway throughput.

Although engineering strategies such as ACC overexpression, orthogonal malonate assimilation pathways, and the non-carboxylative malonyl-CoA (NCM) pathway have substantially increased precursor supply, they cannot completely overcome the regulatory architecture governing central metabolism [57,70,71,72,73,74]. Similarly, dynamic regulatory systems based on malonyl-CoA-responsive biosensors, including FapR-dependent circuits, improve carbon partitioning but remain constrained by sensor dynamic range, response kinetics, and regulatory burden during large-scale fermentation [78,79]. These findings suggest that precursor limitation reflects the intrinsic rigidity of cellular metabolism rather than insufficient expression of individual biosynthetic enzymes. Accordingly, future engineering efforts should integrate orthogonal precursor biosynthesis, dynamic carbon allocation, and growth-production decoupling to achieve more flexible redistribution of metabolic resources instead of relying solely on reinforcement of native malonyl-CoA synthesis.

#### 4.6.5. Product Toxicity and Instability of Pathway Intermediates

The physicochemical properties of anthraquinone metabolites introduce a further layer of complexity into microbial production. Aromatic polyketides frequently possess antimicrobial or cytotoxic activities, suggesting that intracellular accumulation of pathway intermediates or final products may impair host physiology and reduce biosynthetic capacity [48,100,101]. More importantly, several intermediates, particularly flavokermesic acid anthrone, are chemically unstable and readily undergo spontaneous oxidation or polymerization before enzymatic conversion can occur, thereby decreasing effective pathway flux [28]. The importance of intermediate stability is highlighted by recent work in *Y. lipolytica*, where supplementation of fed-batch fermentation with ascorbic acid increased CA production from 3.5 to 9.6 mg L^−1^, representing nearly a threefold improvement [65].

This remarkable enhancement indicates that oxidative degradation of pathway intermediates may represent an underappreciated bottleneck in CA biosynthesis. Future studies should therefore place greater emphasis on protecting reactive intermediates through intracellular redox engineering, antioxidant-assisted fermentation, metabolite channeling, and subcellular compartmentalization, thereby preserving pathway intermediates and improving effective carbon flux.

#### 4.6.6. Product Toxicity, Intermediate Stability, and Transport Engineering

Anthraquinone metabolites exhibit inherent antimicrobial and cytotoxic activities due to their planar aromatic structure, which can intercalate into DNA, disrupt cell membrane integrity, and inhibit respiratory chain function [51]. Intracellular accumulation of CA and its intermediates may therefore impair host physiology, reduce biomass yield, and ultimately limit production titers during prolonged fermentation. More critically, reactive pathway intermediates—particularly flavokermesic acid anthrone—are chemically unstable and prone to spontaneous oxidation and non-enzymatic polymerization under aerobic conditions, leading to carbon loss and the formation of toxic byproducts that induce cellular oxidative stress [28]. The importance of intermediate stability is directly demonstrated by the nearly 3-fold titer improvement (from 3.5 to 9.6 mg/L) achieved by ascorbic acid supplementation in *Y. lipolytica* fed-batch fermentation, confirming that oxidative degradation of intermediates represents a major hidden bottleneck [67]. Two complementary strategies can mitigate these constraints. First, intracellular redox engineering and antioxidant supplementation can protect reactive intermediates from degradation, while subcellular compartmentalization can sequester toxic intermediates away from cytosolic components [37]. Second, efflux transport engineering represents an underexplored approach for CA production. Overexpression of ATP-binding cassette (ABC) transporters or major facilitator superfamily (MFS) transporters can promote extracellular secretion of CA, reducing intracellular product accumulation, alleviating cytotoxicity, and simplifying downstream recovery [31]. To date, systematic transport engineering has not been applied to CA biosynthetic pathways, representing a promising direction for future titer improvement.

## 5. Extraction and Purification of Carminic Acid

Traditionally, CA is industrially produced through extraction from scale insects, primarily *D. coccus* Costa. The extraction and purification processes represent critical downstream steps that fundamentally determine product yield, purity, safety profile, and overall production cost. Conventional extraction methods suffer from inherent limitations such as long processing times, poor target selectivity, excessive organic solvent consumption, high energy input, and variable extraction efficiency across batches. In recent years, advanced extraction technologies and rational purification strategies have been developed to overcome these drawbacks, aiming to achieve higher recovery yields, superior product quality, reduced environmental footprint, and improved process scalability [102]. The downstream processing challenge is not unique to CA but represents a common bottleneck across the natural pigment industry, where recovery and purification can account for up to 60–80% of total production costs [2]. This chapter provides a comprehensive overview of current extraction pathways and purification strategies for CA from both natural insect sources and emerging biotechnological production systems, with particular emphasis on their mechanistic principles, process parameters, and industrial applicability. As consumer demand for clean-label and sustainably produced food ingredients continues to rise, the development of efficient and eco-friendly downstream processing technologies has become increasingly important for the commercial viability of natural colorants [13].

However, the optimal downstream strategy depends strongly on the production platform. For insect-derived CA, solvent extraction combined with adsorption-based purification remains the industrial benchmark due to its robustness and scalability. In contrast, microbial production requires downstream processes adapted to dilute fermentation broths, where cell removal, product recovery, concentration, and impurity removal represent major engineering challenges. Therefore, direct comparison of extraction efficiency between insect biomass and microbial fermentation systems should be avoided because their feedstock matrices and product concentrations differ fundamentally.

### 5.1. Conventional Solvent Extraction

Solvent extraction (SE) remains the most widely explored and industrially employed method for CA recovery from cochineal insects. The underlying principle involves dissolving the target pigment from insect biomass using appropriate solvent systems, followed by solid–liquid separation to remove residual insoluble materials. The selection of solvent composition, extraction temperature, contact time, and solid-to-liquid ratio significantly influences extraction efficiency, product purity, and the overall economics of the process [22]. From a mass transfer perspective, solvent extraction efficiency is governed by the equilibrium solubility of CA in the solvent phase, the diffusivity of solvent into insect tissues, and the disruption of cellular structures that sequester the pigment [20]. The historical development of cochineal extraction technology spans several centuries, evolving from simple aqueous decoction methods used by pre-Columbian civilizations to sophisticated multi-stage solvent extraction processes employed by modern manufacturers [12].

Methanol–water mixtures are among the most effective solvent systems for CA extraction. Systematic optimization studies have shown that a methanol:water ratio of 60:40 (*v*/*v*) can achieve CA concentrations of approximately 370 mg/L under optimized conditions. Acidified solvent systems, particularly those containing tartaric acid or citric acid at concentrations of 0.25–0.5 g/mL, can also yield comparable extraction efficiencies in the range of 368–370 mg/L [22]. Notably, tartaric acid exhibits slightly superior extraction efficacy compared to citric acid, which has been attributed to its stronger chelating capacity and more effective disruption of pigment–protein complexes. The acidification of extraction media not only enhances pigment solubility but also helps stabilize the anthraquinone structure against oxidative degradation during processing [103].

Temperature plays a crucial role in modulating solvent extraction kinetics and equilibrium yield. Generally, increasing temperature within the range of 35–70 °C enhances CA extraction yield, primarily due to improved solvent diffusivity, reduced solvent viscosity, and enhanced pigment solubility in the aqueous–organic phase. However, temperatures exceeding 70 °C fail to further improve extraction performance and may even cause thermal degradation of the pigment, particularly under prolonged exposure. The optimal extraction temperature is typically around 65 °C for most solvent systems, representing a balance between mass transfer enhancement and pigment stability [22,103]. Thermal stability studies have further demonstrated that CA exhibits relatively high heat resistance compared to many other natural pigments, which is consistent with its anthraquinone core structure and C-glycosidic linkage [41]. This inherent thermal stability is a significant advantage for industrial processing, as it allows the use of elevated temperatures to accelerate extraction without substantial product degradation.

Besides methanol–water systems, other solvent combinations have also been investigated for their extraction performance. Ethanol–water mixtures at a 50:50 ratio, followed by liquid–liquid partitioning with ethyl acetate at pH 3, can achieve a CA yield of 52.3%. Methyl isobutyl ketone (MIBK) combined with hot water extraction has been reported to yield up to 57.3%, while conventional hot water extraction alone yields approximately 44.0% [104]. The variation in extraction efficiency across different solvent systems reflects the interplay between solvent polarity, pigment solubility, and the selective dissolution of co-extracted impurities. Traditional hydrochloric acid extraction, although widely used historically, produces relatively low yields, with reported concentrations of only about 0.5 μg/mL under typical conditions, reflecting its limited capacity for efficient pigment solubilization [103].

The traditional commercial manufacturing process for carmine typically achieves an overall yield of approximately 22%, depending on manufacturer proficiency and raw material quality. These processes can be operated in either batch or continuous mode, employing elevated temperatures of 90–100 °C in aqueous environments to maximize pigment release. Pulverizing dried cochineal insects prior to extraction can enhance extraction yields by increasing surface area and facilitating solvent penetration, although it subsequently increases the complexity of the downstream purification process due to the release of finer particulate matter and additional impurities [20]. The quality and composition of cochineal raw material also vary significantly with geographical origin, insect developmental stage, and cultivation conditions, which further contribute to batch-to-batch variability in extraction outcomes [103]. Multivariate statistical analysis of pigment composition has been proposed as a tool for geographical origin characterization, which can help standardize raw material quality and ensure consistent extraction performance [103].

### 5.2. Advanced Extraction Technologies

To address the limitations of conventional solvent extraction—such as high solvent consumption, long processing times, potential thermal degradation, and environmental concerns associated with organic waste disposal—several advanced extraction technologies have been developed and applied to CA recovery. These emerging methods offer improved extraction efficiency, reduced solvent usage, enhanced target selectivity, shorter processing times, and lower energy consumption, aligning with the growing demand for green and sustainable bioprocessing technologies. The development of advanced extraction technologies has been driven not only by economic considerations but also by increasingly stringent environmental regulations and consumer demand for sustainably produced natural products [2].

#### 5.2.1. Pressurized Liquid Extraction

Pressurized liquid extraction (PLE), also known as accelerated solvent extraction (ASE), operates on the principle of performing extraction at elevated temperatures and pressures while maintaining the solvent in its liquid state throughout the process. This technique enables significantly faster extraction compared to conventional methods, while providing enhanced extraction yields with considerably reduced solvent volumes [105]. The elevated pressure increases the boiling point of the solvent, allowing extraction to be performed at temperatures above the normal boiling point without solvent vaporization, thereby enhancing solubility and mass transfer rates.

For CA extraction from cochineal, PLE has demonstrated markedly superior performance compared to traditional solid–liquid extraction. Under optimized conditions with pressures up to 10.5 MPa and temperatures ranging from 100 to 200 °C, PLE can achieve CA yields of up to 42.4%, which is substantially higher than the 18.5% yield obtained through conventional solid–liquid extraction. The extraction time is typically around 30 min, representing a significant reduction in processing duration compared to conventional batch extraction that often requires several hours. Various solvent compositions have been tested for PLE, including methanol:water, ethanol:water, and pure ethanol, each exhibiting different extraction efficiencies depending on operating temperature and pressure conditions [22]. The ability to use ethanol–water mixtures as extraction solvents further enhances the environmental compatibility of PLE, as ethanol is generally recognized as safe (GRAS) and more readily biodegradable compared to methanol.

PLE represents an environmentally friendly extraction technology that combines high efficiency with reduced solvent consumption. Its ability to handle solid samples directly and achieve rapid extraction makes it particularly attractive for industrial-scale applications, although the initial equipment investment may be higher than conventional setups. Beyond CA, PLE has been successfully applied to the extraction of various bioactive compounds from natural sources, including antioxidants from microalgae, further validating its versatility and robustness as an advanced extraction platform [105]. The broader application of PLE across different natural product sectors—including microalgal pigments, plant polyphenols, and functional ingredients—demonstrates its general applicability and potential for widespread industrial adoption [9]. As microalgal biotechnology continues to expand as a source of natural pigments and high-value products, PLE and similar advanced extraction technologies are expected to play an increasingly important role in downstream processing [11].

#### 5.2.2. Supercritical Fluid Extraction

Supercritical fluid extraction (SFE) represents another advanced extraction technology that has gained increasing attention for CA recovery and natural product extraction in general. Typically using carbon dioxide (CO_2_) as the supercritical fluid, SFE offers several inherent advantages including non-toxicity, ease of solvent removal by simple depressurization, reduced environmental impact, and high selectivity for target compounds [106]. CO_2_ is particularly attractive as a supercritical solvent due to its moderate critical parameters (31.1 °C, 7.38 MPa), chemical inertness, non-flammability, and availability at high purity at relatively low cost. Supercritical fluids possess superior transport properties compared to conventional liquids, including lower viscosity and higher diffusivity, which enable them to readily permeate solid materials and achieve faster extraction kinetics. A key feature of SFE is the ability to modulate fluid density by adjusting pressure and/or temperature, thereby fine-tuning the solvent power of the fluid since solubility is directly correlated with density under supercritical conditions [106]. This tunability allows for selective extraction of target compounds by manipulating the operating parameters, potentially reducing co-extraction of unwanted impurities.

For CA extraction, SFE has been investigated under pressures ranging from 15 to 30 MPa at a constant temperature of 40 °C. Under optimized conditions, SFE can achieve CA yields of up to 39.4%, which is comparable to PLE and significantly higher than conventional extraction methods. Methanol is often used as a co-solvent or modifying agent in SFE to enhance the solubility of polar CA molecules in supercritical CO_2_, which is inherently non-polar and thus a poor solvent for highly polar compounds. Notably, similar CA yields have been observed across the pressure range of 15–30 MPa when methanol is employed as a modifier, suggesting that the co-solvent plays a dominant role in solubilizing the pigment [22]. Both PLE and SFE exhibit remarkable selectivity and enable expedited extraction times. Furthermore, the addition of citric or tartaric acid at concentrations exceeding 0.25 g/L has demonstrated pronounced efficacy in the complete removal of proteins from the extracts, which is crucial for reducing allergenic potential in the final product [22]. The removal of insect-derived proteins is of particular importance for food and cosmetic applications, as residual proteins have been identified as a major cause of allergic reactions to cochineal-derived colorants [17,107]. Clinical studies have documented cases of immediate hypersensitivity reactions to cochineal dyes, with protein contaminants recognized as the primary allergenic agents, underscoring the importance of effective protein removal during extraction and purification [17].

### 5.3. Adsorption-Based Purification Strategies

Following initial extraction, CA typically requires further purification to meet the stringent quality requirements for food, cosmetic, and pharmaceutical applications. Crude extracts often contain a complex mixture of impurities including insect proteins, lipids, waxes, other anthraquinone derivatives, and various low-molecular-weight compounds. Adsorption-based purification strategies have emerged as effective and versatile approaches for the selective recovery, concentration, and purification of CA from complex crude extracts. Finite bath adsorption techniques offer the advantage of being able to handle raw sources containing suspended particles, whether organic (biological) or inorganic, without the need for prior clarification or filtration. This enables the simultaneous concentration and selective isolation of the target product from raw extracts or fermentation broths, resulting in higher overall yields and improved cost efficiency by reducing the number of processing steps [108]. The direct processing of unclarified feedstocks is particularly advantageous for industrial-scale operations, as it eliminates the need for expensive and time-consuming pre-treatment steps such as centrifugation or microfiltration.

The adsorptive process involves capturing CA onto high-surface-area solid phases such as porous beads, modified membranes, or nanosized adsorbents. By functionalizing these surfaces with appropriate ligands, the differences in physicochemical properties between the target product and undesirable impurities present in the feedstock can be effectively exploited. Various adsorbent materials have been evaluated for CA recovery, including dextran, agarose, styrene- or acrylic-divinylbenzene resins, silica, and ceramic composites, each offering distinct advantages in terms of binding capacity, selectivity, chemical stability, and cost [108]. The selection of the appropriate adsorbent depends on multiple factors including the nature of the feedstock, target purity requirements, process scale, and economic considerations. Highly specific beaded particles, such as molecularly imprinted polymers (MIPs), represent an advanced approach for CA purification. These imprinted polymers are designed with specific recognition sites tailored to the three-dimensional structure and functional groups of the CA molecule, enabling highly selective binding and recovery. This approach allows for direct recovery of CA from crude cochineal extracts, even in the presence of biological suspended matter and structurally related anthraquinone impurities [109]. Molecular imprinting technology offers the advantage of creating synthetic receptors with antibody-like specificity but with superior mechanical and chemical stability, making them well-suited for repeated use in industrial purification processes.

The structural characteristics of CA, particularly its C-glycosidic linkage and multi-hydroxyl substitution pattern, play important roles in determining its adsorption behavior and purification characteristics. Unlike O-glycosylated anthraquinones, which are more prone to hydrolysis and structural degradation during processing, the C-glycosidic bond confers exceptional chemical stability, allowing the use of a wider range of purification conditions without product degradation [37]. The glycosyl moiety also significantly enhances water solubility compared to the aglycone form, which affects partitioning behavior during liquid–liquid extraction and adsorption selectivity on different resin types [38]. Understanding the relationship between molecular structure and purification properties is essential for rational design of efficient downstream processing strategies. A significant advantage of adsorption-based purification is its ability to remove a substantial portion of allergenic protein molecules from the crude extract when the right adsorption resin and operating conditions are selected. Additionally, by skipping the traditional formation of lakes through metal ion precipitation, the use of toxic metallic ions is circumvented, resulting in a safer and more environmentally friendly production process. The entire processing scheme ensures the delivery of a high-quality product that adheres to stringent food industry standards and regulatory requirements [108]. The European Food Safety Authority (EFSA) has established strict specifications for CA and carmine products used as food additives, including limits for residual heavy metals and protein content, underscoring the importance of effective purification strategies [107]. High-purity CA products also require careful control of isomeric composition. Natural CA isolated from cochineal exists as an equilibrium mixture of two isomers, and the relative proportions of these isomers can affect the color properties and stability of the final product [39]. Kinetic studies have shown that the equilibration between isomers is temperature- and pH-dependent, which has important implications for purification processes that involve thermal treatments or pH adjustments [40]. Advanced chromatographic techniques, including high-performance liquid chromatography (HPLC) coupled with mass spectrometry, have enabled detailed structural characterization and quantification of these isomeric forms, providing valuable analytical tools for quality control during purification [39].

Traditional purification methods involve the formation of insoluble carmine lakes through precipitation with bivalent metal ions, typically aluminum salts. Based on the intended use, the resulting complexes can be treated with concentrated ethanol to precipitate a “soluble” form of carmine, or isolated in an “insoluble” state below pH 7 through the introduction of calcium salts. While these methods are well-established and have been used for decades, they often lead to variable product quality and may introduce metal ion contaminants that require additional removal steps [20]. Furthermore, the use of metal ions raises concerns about potential toxicity and environmental impact, driving the development of alternative metal-free purification strategies.

### 5.4. Enzyme-Assisted Extraction

Enzyme-assisted extraction represents another promising strategy to improve CA recovery from cochineal biomass. The use of proteolytic enzymes can help break down insect proteins and cellular structures, facilitating the release of CA molecules that may be bound to insoluble fractions or sequestered within protein complexes. This approach leverages the catalytic specificity of enzymes to disrupt biological matrices under mild conditions, potentially reducing energy consumption and minimizing pigment degradation compared to harsh chemical or thermal treatments. Optimized extraction techniques can achieve heightened yields when ground cochineal insects are processed with proteolytic enzymes in aqueous systems, particularly in the presence of appropriate chemical surfactants that further enhance cell disruption and pigment solubilization. For example, neutral protease treatment at 50 °C with stirring for 16 h has been employed to enhance CA release from cochineal biomass. Following enzymatic treatment, further purification can be carried out using ion-exchange chromatography to achieve higher product purity and remove residual enzyme and protein fragments [110]. The combination of enzymatic pre-treatment with chromatographic purification represents an integrated approach that can deliver high-purity CA with good recovery yields.

Enzyme-assisted approaches can also help reduce the amount of residual protein in the final extract, thereby lowering the allergenic potential of the product. This is particularly important for food-grade and cosmetic-grade CA products, where protein content is strictly regulated by authorities such as the European Food Safety Authority (EFSA) to minimize the risk of allergic reactions in sensitive individuals [107,110]. Allergic reactions to cochineal-derived colorants have been documented in clinical settings, and protein impurities are generally recognized as the primary causative agents [17]. Therefore, effective protein removal during extraction and purification is not only a quality requirement but also a critical safety consideration.

### 5.5. Downstream Processing of Biotechnologically Produced Carminic Acid

With the emergence of biotechnological production platforms for CA based on microbial cell factories, the development of appropriate downstream processing strategies for microbial fermentation broths has become increasingly important. The extraction and purification methods for microbially produced CA differ significantly from those used for insect-derived material, due to fundamental differences in the starting matrix, product concentration, impurity profiles, and regulatory requirements. Fermentation broths typically contain lower product concentrations but also a different spectrum of impurities, including microbial cells, media components, metabolic byproducts, and pathway intermediates. The challenge of downstream processing for microbially produced pigments is widely recognized across the biotechnology industry, and significant research efforts are being directed toward developing efficient recovery strategies for various pigment classes [2].

Currently, extraction methods for biotechnologically produced CA have been primarily developed at laboratory scale, tailored to specific microbial hosts and their respective cultivation conditions. For *A. nidulans* fermentation, CA extraction involves cell lysis by first concentrating the fungal biomass via centrifugation, followed by resuspension of the pellet in methanol containing 1% formic acid, aided by ultrasonication to disrupt cell walls and release intracellular pigments. The resulting extract is then purified and analyzed using high-performance liquid chromatography coupled with high-resolution tandem mass spectrometry (HPLC-HRMS/MS) for accurate identification and quantification [28]. The choice of extraction solvent and lysis method is critical for fungal systems, as the rigid cell wall can pose a significant barrier to efficient product recovery. For *E. coli*-based production systems, solid-phase extraction (SPE) has been employed for the recovery of fermentation products, followed by purification and analysis using liquid chromatography–mass spectrometry (LC-MS). In fed-batch fermentation reactors producing CA at levels of approximately 0.65 mg/L, this approach enables effective concentration and cleanup of the target compound from the complex fermentation broth matrix [43]. Solid-phase extraction is particularly well-suited for dilute aqueous solutions, as it allows for large volume sample loading and selective retention of the target analyte, resulting in significant concentration factors and simultaneous removal of polar impurities. In *S. cerevisiae* fermentation systems, where CA titers up to 7.6 mg/L have been achieved, extraction is performed using an organic solvent mixture consisting of ethyl acetate, n-butanol, and formic acid at a ratio of 69:30:1 (*v*/*v*/*v*). The organic phase is then evaporated under nitrogen gas flow, and the final product is resuspended in methanol for further HPLC analysis and quantification [29]. The selection of the solvent mixture is based on the polarity of CA and the need to achieve efficient liquid–liquid extraction from aqueous fermentation broth, with formic acid serving to acidify the aqueous phase and promote partitioning of the acidic pigment into the organic phase.

The downstream processing challenges associated with microbially produced CA are not unique but are shared across many natural pigment production platforms. For example, microbial production of anthocyanins—another important class of natural pigments—faces similar challenges including low titers, complex impurity profiles, and the need for efficient recovery and purification strategies [5]. Current bottlenecks in microbial anthocyanin production include not only biosynthetic limitations but also downstream processing constraints, and addressing these challenges requires integrated approaches that combine metabolic engineering with bioprocess optimization [6]. Similarly, microbial production of carotenoids has advanced significantly in recent years, and the downstream processing strategies developed for these pigments can provide valuable insights and transferable technologies for CA recovery [3]. The broader development of sustainable production strategies for natural colorants, encompassing both biosynthetic optimization and downstream processing, is essential for realizing the full potential of microbial cell factories in the food and cosmetic industries [4]. Other microbial pigment systems offer additional perspectives on downstream processing development. For instance, violacein—a violet pigment produced by various bacterial species—has been extensively studied for its microbial synthesis and potential applications, and the purification strategies developed for this pigment provide useful references for CA processing [8]. A comprehensive review of violacein production by microbial fermentation highlights the importance of integrating upstream strain engineering with downstream processing optimization to achieve economically viable production [7]. These cross-pigment comparisons underscore the general principle that efficient downstream processing is a critical enabler for the commercialization of microbial pigment production platforms.

It is important to note that carmine extracts often contain not only CA but also other related anthraquinone compounds generated during the metabolic pathway, such as kermesic acid and flavokermesic acid. Since these compounds also exhibit red pigmentation, they may not need to be removed depending on the final target application, as they can contribute to the overall coloring capacity of the product. However, for food and cosmetic applications where consistent composition and strict quality control are required, thorough purification is essential to remove potential contaminants including proteins and other biologically active molecules that could cause allergic reactions or other adverse effects [102]. The presence of pathway intermediates also provides valuable information about pathway efficiency and can guide further metabolic engineering efforts to improve flux toward the final product. Although current research has primarily focused on the production of CA on chassis, well-developed strategies for the recovery and purification of this secondary metabolite will be essential for successful industrial-scale implementation. These strategies should be specifically designed based on several considerations, including the microorganism involved, fermentation operating conditions, production scale, target product purity, and the type of application for which the CA is intended. As microbial production titers continue to improve through metabolic engineering, the development of scalable and cost-effective downstream processing will become increasingly critical for the economic viability of biotechnological CA production. The integration of next-generation genetic and fermentation technologies with advanced downstream processing approaches will be key to achieving safe, sustainable, and economically competitive production of natural food colorants [13].

A major limitation of current microbial production systems is that reported CA titers remain far below those required for economically competitive industrial fermentation. At laboratory scale, solvent extraction and chromatographic purification are suitable for analytical characterization; however, these approaches are unlikely to be economically feasible for large-scale production due to solvent consumption and processing costs. Future industrial processes will require integrated downstream strategies involving in situ product recovery, adsorption-based capture, membrane-assisted concentration, or continuous purification technologies to reduce downstream expenditure. No comprehensive techno-economic assessment has yet established the exact production titer required for microbial CA to outperform conventional insect extraction. However, competitiveness will depend not only on fermentation titer but also on substrate cost, fermentation productivity, downstream recovery efficiency, and regulatory acceptance.

Based on current evidence, adsorption-assisted purification combined with mild extraction conditions appears to represent the most practical strategy for preserving CA stability while achieving high recovery. Nevertheless, most optimization studies have been performed using insect-derived material, and dedicated downstream processes for fermentation-derived CA remain insufficiently explored. A systematic comparison of these strategies is summarized in Table 4.

For insect-derived CA, pressurized liquid extraction combined with macroporous adsorption resin purification currently represents the optimal integrated strategy, balancing recovery yield, protein removal efficiency, and process scalability. Acidified PLE achieves higher extraction efficiency than conventional solvent extraction with reduced solvent usage, and subsequent adsorption purification effectively removes residual insect proteins—the primary allergenic components—while concentrating the pigment [22,108]. For microbially produced CA, given the low product concentration in fermentation broths (mg/L level), the most industrially viable downstream route is expected to be cell removal followed by adsorptive capture and concentration, and final crystallization as a polishing step. Current laboratory-scale protocols rely on organic solvent liquid–liquid extraction, which is not economically feasible at industrial scale due to high solvent cost and energy consumption. Membrane separation and in situ product recovery are emerging technologies that could reduce downstream costs, but their application to CA production requires further validation.

## 6. Anthraquinone Pigments Structurally Related to Carminic Acid

CA belongs to a diverse family of anthraquinone pigments that share a conserved tricyclic aromatic scaffold but exhibit substantial variation in substitution patterns, glycosylation modes, and biosynthetic origins. This structural diversity underpins a wide range of physicochemical properties and biological functions, providing a broader context for understanding CA biosynthesis and for guiding pathway engineering efforts [45,48].

### 6.1. Structural Diversity and Functional Implications

Anthraquinones are defined by a planar three-ring core that is variably decorated with hydroxyl, methyl, and glycosyl substituents. Within this framework, relatively small modifications can lead to pronounced changes in chemical stability, solubility, and bioactivity [45,46]. A distinguishing feature of CA is its C-glycosidic linkage, which confers exceptional resistance to hydrolysis and thermal degradation compared to the more common O-glycosylated anthraquinones found in plants. This structural trait highlights glycosylation mode as a critical determinant of pigment stability and industrial applicability [37,47,52].

**Figure 4:**
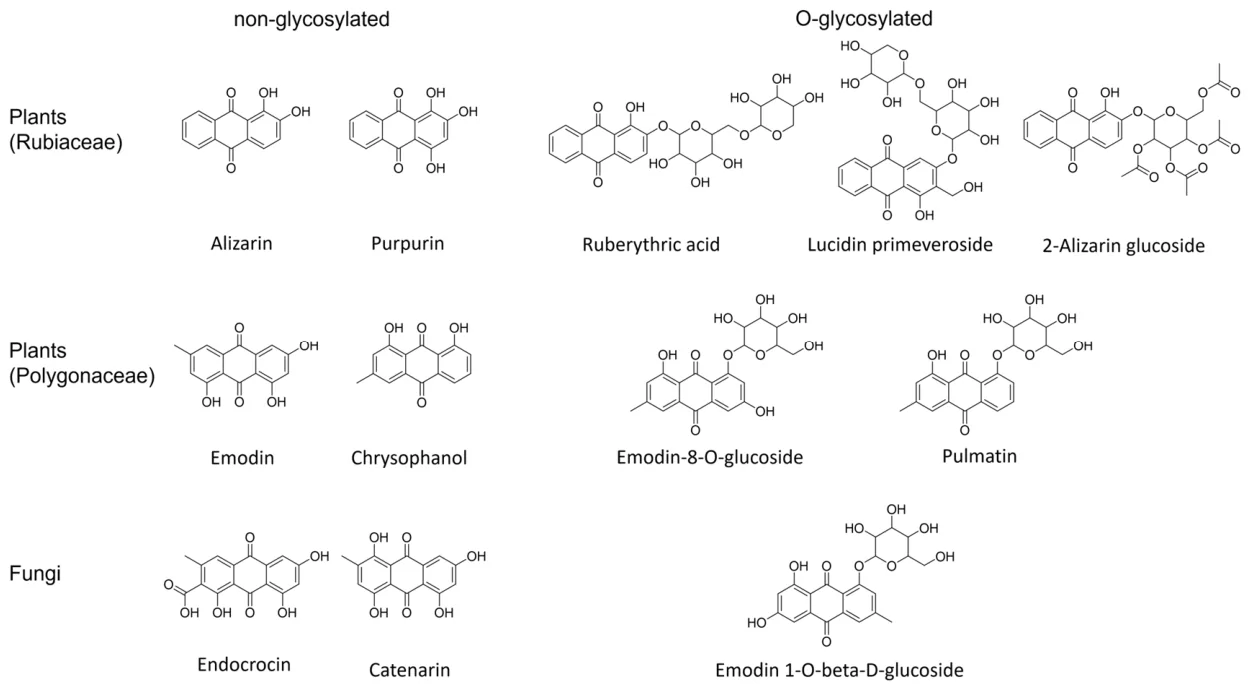
Representative non-glycosylated and O-glycosylated anthraquinones from plants and fungi, highlighting the diversity of anthraquinone glycosylation compared with the C-glycosylated structure of carminic acid.

The structural relationships among CA and related anthraquinone analogues are illustrated in Figure 4. Non-glycosylated anthraquinones such as alizarin and purpurin illustrate how hydroxylation patterns modulate electronic structure and, consequently, color properties and metal-chelating capacity. In parallel, emodin-type anthraquinones represent a structurally distinct class characterized by substitutions on both terminal rings, reflecting alternative biosynthetic tailoring. Beyond plants, bacteria produce highly modified anthraquinones with expanded ring systems and fused scaffolds, including anthraquinone–γ-pyrones and enediyne-associated derivatives. These structures often exhibit potent bioactivities, underscoring the capacity of microbial metabolism to generate chemically complex anthraquinone architectures [42,46,48,54,111]. Collectively, these variations demonstrate that functional diversification within the anthraquinone family is primarily driven by tailoring reactions acting on a conserved core scaffold.

### 6.2. Divergent Biosynthetic Strategies

Anthraquinone biosynthesis has evolved through distinct metabolic routes across kingdoms, most notably the polyketide pathway and the shikimate-derived pathway [34,112]. The polyketide route, which underlies CA biosynthesis, relies on iterative condensation of malonyl-CoA units to generate an octaketide intermediate that subsequently cyclizes into the anthraquinone core. This mechanism is also responsible for the formation of emodin-type anthraquinones and highlights the central role of type III polyketide synthases in constructing the scaffold [14,45,48,61,113].

In contrast, many plant-derived anthraquinones, including alizarin and purpurin, originate from the shikimate pathway, where the aromatic rings are assembled through intermediates such as chorismate and o-succinylbenzoate, followed by prenylation and cyclization reactions. The activation of o-succinylbenzoate to its CoA ester is catalyzed by a dedicated ligase that generates ADP rather than AMP, a mechanistic feature distinct from bacterial homologs. The prenylation of 1,4-dihydroxy-2-naphthoic acid by membrane-bound prenyltransferases represents the first committed step in this route, channeling intermediates toward the formation of alizarin-type structures [34,45,112].

Downstream diversification is largely governed by tailoring enzymes, including hydroxylases, prenyltransferases, and glycosyltransferases. In particular, the distinction between O- and C-glycosylation represents a key branching point that significantly influences molecular stability and function. Structural studies of glycosyltransferases have revealed conserved folds but flexible active sites, enabling both substrate promiscuity and evolvability, as demonstrated by the ability of certain plant O-glycosyltransferases to accept multiple sugar donors and to be redirected toward new substrates through single-residue mutations [27,47,55]. Beyond the well-characterized Rubiaceae anthraquinone pathways, transcriptomic approaches have facilitated the discovery of new biosynthetic genes in related species. For example, de novo transcriptome analysis of Rubia yunnanensis has identified putative genes involved in anthraquinone biosynthesis, providing valuable genetic resources for pathway elucidation and enzyme discovery [114]. Similarly, the identification and characterization of glycosyltransferases from other medicinal plants, such as three 6-hydroxyalizarin glycosyltransferases from Rheum palmatum, have further expanded the repertoire of anthraquinone-modifying enzymes available for synthetic biology applications [115].

These observations highlight that anthraquinone biosynthesis is a product of convergent evolution, in which distinct metabolic frameworks generate structurally similar end products through different biochemical logics.

### 6.3. Structure–Activity Relationships and Biological Functions

The biological activities of anthraquinones are closely linked to their planar aromatic structure and substitution patterns. The conjugated core enables π–π interactions with biomolecules such as DNA, forming the basis for activities including intercalation and topoisomerase inhibition [45,46,111]. Hydroxylation patterns play a central role in modulating redox behavior and metal-binding capacity, thereby influencing antioxidant and enzyme inhibitory activities. For instance, the 1,2,4-trihydroxy arrangement found in purpurin confers both radical-stabilizing capacity and monoamine oxidase inhibition, demonstrating how specific hydroxylation patterns can enable distinct pharmacological functions. Similarly, the number and position of functional groups determine antimicrobial potency, with particular substitution patterns enhancing interactions with bacterial targets [46,48]. Glycosylation introduces an additional layer of functional modulation. While C-glycosylation enhances chemical stability, O-glycosylation generally improves solubility and alters bioavailability [100]. These modifications illustrate how tailoring reactions fine-tune both physicochemical properties and biological activity [47,52].

However, the bioactivity of anthraquinones is often accompanied by toxicity concerns. Long-term exposure to certain hydroxyanthraquinones has been associated with nephrotoxicity and carcinogenicity in animal models, underscoring the importance of structural optimization and dose control when developing anthraquinone-based applications [46,111]. Beyond their well-documented pharmacological activities, anthraquinones have also been recognized for their diverse health benefits spanning multiple therapeutic areas. Multiple comprehensive reviews systematically sort the therapeutic potential of anthraquinone natural products [101,111]. Their laxative, antitumor, antimalarial, antibacterial, antifungal, and antioxidant properties have been extensively reviewed, highlighting their broad potential as bioactive scaffolds for drug development and functional applications [54,111].

### 6.4. Implications for Synthetic Biology and Metabolic Engineering

The expanding understanding of anthraquinone biosynthesis provides a framework for engineering microbial production systems. The diversity of natural pathways offers a rich toolkit of enzymes that can be recombined to construct synthetic routes with tailored properties [1]. Glycosyltransferases are of particular interest due to their substrate promiscuity and tunable specificity. Structure-guided engineering has demonstrated that minor modifications in active-site residues can significantly alter regioselectivity and catalytic scope, enabling the conversion of O-glycosyltransferases into C-glycosyltransferases or the expansion of sugar donor selectivity. These enzymes therefore represent key leverage points for pathway diversification [27,47,51,115]. In addition, transcriptomic and pathway discovery approaches continue to identify new biosynthetic genes and regulatory elements, expanding the repertoire of components available for engineering. The integration of enzymes from different organisms—including plants, bacteria, and insects—further enables the construction of modular and hybrid pathways that do not exist in nature, allowing access to both known anthraquinones and novel derivatives [26,43,114].

These advances suggest that anthraquinone biosynthesis is highly amenable to synthetic biology, with opportunities not only for improved production of established compounds but also for the creation of derivatives with enhanced stability, solubility, or bioactivity.

### 6.5. Outlook: Toward Engineered Anthraquinone Platforms

Despite significant progress, several challenges remain in translating anthraquinone biosynthesis into practical applications. Limitations in solubility, stability, and bioavailability continue to constrain their use, particularly in therapeutic contexts. The poor systemic exposure of certain anthraquinones following oral administration highlights the need for improved formulation strategies or metabolic engineering to enhance pharmacokinetic properties [1,46]. Toxicity associated with certain substitution patterns also necessitates careful evaluation. The long-term safety profiles of anthraquinones vary considerably with structural features, reinforcing the importance of targeted engineering to separate desired bioactivity from adverse effects [48]. From a production perspective, plant-based systems remain an important source of anthraquinones, and strategies for enhancing their yield in plant cell and organ cultures continue to be developed. In vitro elicitation approaches, including precursor feeding and elicitor treatment, have been widely explored to boost anthraquinone accumulation in callus and cell suspension cultures [116]. Similarly, optimization of adventitious root culture conditions, including auxin concentrations, elicitors, and precursor supplementation, has been shown to significantly increase the content of alizarin and purpurin in Rubia cordifolia, providing alternative production platforms that complement microbial biosynthesis [117].

Future efforts will likely focus on integrated engineering strategies that combine pathway reconstruction, enzyme optimization, and process design. The development of modular platforms capable of producing diverse anthraquinone derivatives—coupled with advances in formulation and delivery—will further expand their application space in both industrial and pharmaceutical contexts. Overall, the study of structurally related anthraquinones not only provides insight into the biosynthetic logic of CA, but also establishes a broader foundation for the design of functionalized aromatic polyketides in engineered systems.

## 7. Conclusions and Perspectives

CA is a distinctive natural anthraquinone pigment defined by its cross-kingdom biosynthetic logic, which merges plant-like type III polyketide scaffold assembly with insect-specific enzymatic tailoring—most notably the membrane-associated C-glycosylation step. Over the past decade, advances in insect transcriptomics, heterologous pathway reconstruction, and synthetic biology have progressively resolved the core enzymatic framework of CA biosynthesis and established de novo production in four representative microbial chassis: *E. coli*, *S. cerevisiae*, *Y. lipolytica*, and *A. nidulans*.

Quantitative cross-chassis comparison across published studies reveals a consistent hierarchy of engineering contributions: fermentation process optimization delivers the largest relative titer gains (5–8-fold), followed by precursor supply engineering (1.7–3.4-fold), while single-enzyme modifications typically yield incremental improvements (<2-fold). Strategies including malonyl-CoA pool expansion, peroxisomal compartmentalization, and antioxidant-assisted fermentation have all proven effective in boosting production, yet the highest reported titer remains at 9.6 mg/L in fed-batch culture of engineered *Y. lipolytica* [67]—still orders of magnitude below the gram-per-liter threshold typically required for industrial cost competitiveness.

### 7.1. Core Challenges Limiting Production Scale-Up

The modest performance of microbial CA cannot be attributed to a single rate-limiting enzyme, but arises from interconnected constraints across molecular, pathway, and cellular levels. First, the reconstructed pathway suffers from intrinsic catalytic inefficiency. The type III PKS octaketide synthase generates multiple shunt products due to spontaneous cyclization of the highly reactive linear octaketide intermediate, dissipating most carbon flux before it enters the productive anthraquinone route [28]. The terminal C-glycosylation step, catalyzed by the endoplasmic reticulum-bound insect enzyme DcUGT2, imposes a further catalytic bottleneck, and its functional expression remains incompatible with prokaryotic hosts [55]. Second, cross-kingdom enzyme incompatibility undermines pathway fidelity. The current hybrid pathway assembles enzymes from plants, bacteria, fungi, and insects within a single microbial host, leading to suboptimal protein folding, mismatched cofactor requirements, and impaired inter-enzyme coordination. This necessitates extensive pathway rewiring in prokaryotic chassis, further reducing catalytic efficiency.

Third, central metabolic rigidity restricts precursor allocation. Malonyl-CoA is strictly regulated as an essential precursor for fatty acid biosynthesis and cell membrane biogenesis, and even aggressive precursor engineering strategies cannot fully decouple secondary metabolism from essential cellular functions [48]. NADPH cofactor limitation, driven by oxidative tailoring reactions, represents an additional underrecognized metabolic constraint. Fourth, intermediate instability and product toxicity create hidden flux losses. Reactive anthrone intermediates undergo spontaneous oxidation and non-enzymatic polymerization under aerobic conditions, while accumulated anthraquinone metabolites can disrupt membrane integrity and cellular redox homeostasis, limiting sustainable production during extended fermentation [28,51].

### 7.2. Integrated Synthetic Biology Strategies for Breakthrough Titer Improvement

To overcome these multi-layered limitations and push CA titers toward industrially relevant levels, isolated single-target engineering is no longer sufficient. Instead, integrated multi-dimensional strategies combining orthogonal precursor pathways, spatial organization, enzyme engineering, and dynamic flux control are required.

#### 7.2.1. Precursor Pathway Rewiring and Metabolic Decoupling

Traditional acetyl-CoA carboxylase overexpression is fundamentally constrained by native regulatory networks. The emerging non-carboxylative malonyl-CoA (NCM) pathway, which bypasses the ATP-consuming acetyl-CoA carboxylation step and decouples malonyl-CoA synthesis from central carbon metabolism, represents a transformative solution [36]. Combining the NCM pathway with tunable repression of competing fatty acid biosynthesis via CRISPR interference (CRISPRi) [87] will enable high-flux malonyl-CoA supply without compromising cellular fitness. Two-stage fermentation processes that decouple biomass accumulation from product formation can further alleviate the inherent growth-production trade-off. Cell-free biosynthesis systems also serve as a complementary tool for rapid pathway prototyping and enzyme kinetic characterization, decoupling catalytic reactions from cellular growth constraints to accelerate pathway debugging.

#### 7.2.2. Subcellular Compartmentalization and Substrate Channeling

Spatial engineering through organelle compartmentalization offers a powerful strategy to sequester pathway intermediates, reduce competing side reactions, and increase local concentrations of enzymes and substrates [37]. Peroxisomal targeting of the CA biosynthetic pathway, validated to improve titer by 27% in *Y. lipolytica* [67], can be further optimized by expanding peroxisome capacity via machine learning-guided engineering [95]. In addition, construction of artificial enzyme complexes via synthetic scaffolding or protein fusion can enable substrate channeling between consecutive pathway steps, reducing intermediate leakage and improving overall pathway throughput [96].

#### 7.2.3. AI-Assisted Enzyme Engineering for Enhanced Catalytic Fidelity

To date, no AI-designed enzyme specifically optimized for CA biosynthesis has been experimentally validated, and this remains a high-priority future direction. AlphaFold-driven structural modeling has provided near-atomic resolution structural models for OKS and DcUGT2, laying a solid foundation for rational active-site remodeling [90,91]. Machine learning-guided protein fitness prediction can further accelerate directed evolution campaigns [93], with two primary optimization goals: first, engineering OKS variants with enhanced cyclization fidelity to reduce shunt product formation; second, engineering soluble, highly active C-glycosyltransferase variants to overcome the membrane-dependence bottleneck and enable functional expression in prokaryotic chassis. AI-assisted enzyme engineering is expected to address the root cause of intrinsic pathway inefficiency and deliver step-change improvements in carbon yield [38].

#### 7.2.4. Biosensor-Based Dynamic Regulation and Systems-Level Flux Balancing

Long multi-enzyme pathways such as CA biosynthesis exhibit distributed flux control, where overexpression of a single enzyme often leads to bottleneck migration and diminishing returns. Genetically encoded malonyl-CoA biosensors (e.g., FapR-based circuits) enable hierarchical dynamic regulation that autonomously coordinates pathway enzyme expression with cellular metabolic state [77,81]. Coupling these biosensors with multi-layered regulatory networks can dynamically repress competing pathways and activate biosynthetic genes when precursor availability is sufficient, achieving optimal flux allocation without manual intervention. Genome-scale metabolic models (GEMs) can further guide systematic target identification, complementing experimental engineering with computational prediction [31]. Adaptive laboratory evolution (ALE) provides an additional complementary approach to select for strains with enhanced precursor supply and product tolerance.

#### 7.2.5. Transport Engineering and Cytotoxicity Mitigation

Efflux transport engineering remains a largely underexplored avenue for CA production optimization. Overexpression of ATP-binding cassette (ABC) or major facilitator superfamily (MFS) transporters can promote extracellular secretion of CA and its intermediates, reducing intracellular accumulation, alleviating anthraquinone-induced cytotoxicity, and simplifying downstream recovery [31]. Complementary strategies including intracellular redox homeostasis engineering and antioxidant supplementation can protect reactive anthrone intermediates from oxidative degradation, as demonstrated by the nearly 3-fold titer improvement from ascorbic acid addition in *Y. lipolytica* fed-batch fermentation [67].

### 7.3. Industrial Translation: Regulatory, Economic and Sustainability Considerations

Beyond strain engineering, successful industrialization of microbial CA requires systematic consideration of regulatory compliance, economic viability, and verifiable sustainability.

#### 7.3.1. Regulatory Status and Compliance Pathways

Currently, CA is approved as food additive E120 by major regulatory bodies including EFSA and FDA, but all existing approvals are based on insect-derived raw materials [107]. Microbially produced CA will follow distinct regulatory frameworks in different jurisdictions: in the European Union, it will require Novel Food authorization; in the United States, manufacturers can pursue Generally Recognized As Safe (GRAS) status. Microbial production offers an inherent safety advantage in allergen control, as it eliminates insect protein contaminants that are the primary cause of documented cochineal dye-induced immediate hypersensitivity reactions [17]. However, strict quality controls on residual host DNA, endotoxins, and pathway intermediate impurities will be required to meet food and pharmaceutical grade specifications. Harmonization of quality standards with existing insect-derived CA products will be critical for smooth market acceptance [107,118].

#### 7.3.2. Techno-Economic Outlook and Market Entry Roadmap

To date, no comprehensive techno-economic assessment (TEA) for microbially produced CA has been published. For reference, industrial insect-extracted food- and cosmetic-grade CA is priced at 120–240 USD per kilogram, with production costs dominated by insect cultivation, harvesting, and downstream purification [12]. For microbial fermentation to achieve cost parity with insect extraction, titers generally need to reach the gram-per-liter level for bulk natural products, meaning current production levels require at least 100-fold improvement. The cost structure of microbial production will be dominated by fermentation substrates and downstream processing, with downstream recovery accounting for 60–80% of total production costs for dilute pigment fermentation broths [2].

In the near term, microbial CA is most likely to first penetrate high-value niche markets such as allergen-free pharmaceutical colorants and premium cosmetics, where higher price points can offset low production volumes. Broad market competitiveness with traditional insect extraction will require concurrent advances in fermentation titer, volumetric productivity, and low-cost downstream recovery technologies such as adsorptive capture and in situ product removal.

#### 7.3.3. Sustainability Verification and Green Manufacturing

Microbial fermentation is widely regarded as a promising sustainable alternative to traditional insect farming, as it eliminates dependence on Opuntia cactus cultivation and tropical climatic conditions, reduces land use intensity, and enables year-round production under fully controlled conditions [1,2]. However, quantitative life-cycle assessment (LCA) and carbon footprint data directly comparing the two production routes are currently lacking. The actual sustainability performance of microbial CA will depend heavily on the adoption of renewable carbon substrates (e.g., lignocellulosic biomass, industrial waste streams), low-carbon energy for fermentation, and green downstream processes. Future research should integrate LCA into early-stage process development to ensure that sustainability claims are supported by empirical data.

### 7.4. Toward a Versatile Anthraquinone Synthetic Biology Platform

The development of CA microbial cell factories also establishes a foundational platform for the biosynthesis of diverse anthraquinone pigments and bioactive derivatives. The anthraquinone family exhibits enormous structural diversity, with variations in hydroxylation patterns, methylation, and glycosylation modes giving rise to a broad spectrum of color properties and biological activities [35,51]. The modular CA biosynthetic pathway can be readily diversified by swapping tailoring enzymes: substituting hydroxylases, methyltransferases, or glycosyltransferases from plant, fungal, or bacterial sources can generate both known anthraquinone compounds and new-to-nature derivatives with tailored stability, solubility, or bioactivity. In particular, expanding the substrate scope of C-glycosyltransferases to diverse anthraquinone scaffolds can create a new class of highly stable C-glycosylated anthraquinone pigments with superior industrial performance compared to conventional O-glycosylated counterparts.

Looking ahead, the combination of pathway modularity, expanding enzyme toolboxes, and AI-guided molecular design will transform anthraquinone biosynthesis from case-by-case pathway reconstruction into a programmable synthetic biology platform, enabling on-demand production of high-value pigments and pharmaceutical intermediates.

### 7.5. Final Outlook

In conclusion, microbial biosynthesis of CA has advanced from initial pathway elucidation to proof-of-concept production in multiple microbial chassis, demonstrating the technical feasibility of sustainable manufacturing of this cross-kingdom natural product. While significant scientific and engineering challenges remain before large-scale industrialization is achievable, the convergence of synthetic biology, protein engineering, and bioprocess technology provides a clear roadmap for future breakthroughs. As research progresses, microbial CA and expanded anthraquinone platforms will not only address the supply limitations and safety concerns of traditional natural colorants, but also open new avenues for the design of functional biobased pigments, bridging fundamental natural product research with industrial green biomanufacturing.

## Figures and Tables

**Table 1:** Enzymes involved in the heterologous synthesis pathway of carminic acid.

Biosynthetic Step	Enzyme	EC Classification	Source Organism	Catalytic Function	Experimental Evidence	Current Characterization
Octaketide formation	OKS (AaOKS)	EC:2.3.1.- (putative)	*Aloe arborescens*	Condensation of acetyl-CoA and seven malonyl-CoA units to generate linear precursor	Heterologous expression and metabolite identification in engineered hosts	Experimentally validated; surrogate enzyme rather than native insect enzyme
First cyclization	ZhuI	Not assigned	*Streptomyces* sp. R1128	Regioselective cyclization of octaketide intermediate	Functional reconstruction in heterologous pathway	Functionally validated surrogate enzyme
Aromatization	ZhuJ	Not assigned	*Streptomyces* sp. R1128	Aromatization and structural rearrangement of cyclized intermediate	Functional reconstruction in heterologous pathway	Functionally validated surrogate enzyme
C-glycosylation	DcUGT2	EC:2.4.1.- (putative)	*D. coccus*	C-glucosylation of flavokermesic acid and kermesic acid using UDP-glucose	Transcriptomic identification, biochemical assays, and heterologous expression	Biochemically characterized native insect enzyme
Anthrone oxidation	AptC	EC:1.14.14.- (putative)	*A. nidulans*	Oxidation of flavokermesic acid anthrone to flavokermesic acid	Candidate enzyme screening and yeast pathway reconstruction	Experimentally validated heterologous enzyme
Hydroxylation	Cat5	EC:1.14.99.60	*S. cerevisiae*	Hydroxylation of flavokermesic acid to kermesic acid	Functional screening and metabolite analysis in engineered yeast	Experimentally validated host-derived enzyme
Alternative C-glycosyltransferases	Plant/microbial CGTs		Plants/ microorganisms	Formation of aromatic C-glycosidic bonds in flavonoid or related substrates	Biochemical characterization and structural studies	Engineering references; direct involvement in CA biosynthesis not established

**Table 2:** Comparison of four heterologous expression hosts.

Host/Feature	*E. coli*	*S. cerevisiae*	*Y. lipolytica*	*A. nidulans*
Growth rate	Very fast	Moderate	Moderate	Slow
Genetic accessibility	Excellent; mature CRISPRi, plasmid expression, abundant promoter libraries	Good; complete gene knockout/overexpression toolkit, eukaryotic modification system	Moderate; fewer standardized regulatory elements than S. cerevisiae	Poor; complex homologous recombination, low-throughput genetic manipulation
Malonyl-CoA availability	Low; strong competition with fatty acid synthesis, tight native metabolic regulation	Medium; limited acetyl-CoA supply from central carbon metabolism	Very high; inherent lipid metabolism provides abundant acetyl-CoA/malonyl-CoA	Medium; endogenous PKS consumes partial malonyl-CoA for native secondary metabolites
Product toxicity tolerance	Low; anthraquinone intermediates inhibit membrane function; unstable octaketide easily degrades	Medium; eukaryotic membrane reduces intermediate leakage, weak oxidative stress response	Medium-high; strong antioxidant capacity, peroxisome compartmentation alleviates toxic exposure	Medium; robust endogenous antioxidant system but mycelium accumulates toxic intermediates
Pathway characteristics	Rewired pathway using surrogate enzymes	More native-like pathway; functional expression of DcUGT2	Enhanced precursor-oriented pathway	Native-like polyketide biosynthesis context
Titer (Batch feeding)	0.63 ± 0.02 mg/L	7.58 mg/L	9.6 mg/L	Only trace qualitative detection; yields were too low to quantify.
Strength	Rapid growth; strong genetic tools; ideal for pathway prototyping	Supports membrane-associated enzymes; enables C-glycosylation	High acetyl-CoA/malonyl-CoA availability; suitable for polyketide synthesis	Endogenous PKS machinery; suitable intracellular environment
Bottleneck	Incompatibility with eukaryotic enzymes; limited tailoring capacitys	Flux imbalance; limited precursor supply	Limited pathway characterization; regulation complexity	Complex genetics; slower growth
Engineering focus	Pathway rewiring; improving precursor supply; enhancing protein expression	Flux balancing; improving acetyl-CoA/malonyl-CoA supply; optimizing localization	Redirecting lipid metabolism; improving pathway integration	Leveraging native systems; improving genetic tools
Industrial scalability	High; cheap carbon source, simple fermentation equipment, easy high-cell-density culture	High; food-grade safety chassis, mature large-scale yeast fermentation process	Very high; oleaginous yeast suitable for low-cost raw materials, high-density fermentation	Low; long fermentation cycle, difficult mycelium separation, high downstream cost

**Table 3:** Carminic acid production titers and engineering strategies across different microbial hosts.

Engineering Phase	*E. coli* (mg/L)	*S. cerevisiae* (mg/L)	*Y. lipolytica* (mg/L)	*A. nidulans* (mg/L)
Initial pathway construction (shake flask method)	0.022	2.66	0.7	Trace
Precursor Supply Engineering (ACC/NCM)	0.075 (3.4×)	—	1.2 (1.7×)	—
β-Oxidation/lipid metabolism engineering	—	4.07 (1.5×)	1.5 (1.25×)	—
Enzyme engineering/fusion proteins	—	4.07 (1.5×)	—	—
Compartmentalization (peroxisomes)	—	—	1.9 (1.27×)	—
Glycosylation optimization	—	3.04 (1.14×)	—	—
Fed-batch fermentation with supplementation (final stage)	0.63	7.58	9.6	Trace
Fold increase from shake-flask to fed-batch fermentation	~8.4×	~1.9×	~5.1×	—

Notes: Fold changes in parentheses represent the increase relative to the corresponding reference strain or preceding engineering stage reported in the original study. Values obtained from independent engineering strategies are not necessarily cumulative.

**Table 4:** Comparison of downstream processing strategies for carminic acid.

Parameter/Strategy	Principle	Typical Recovery Yield	Protein Removal Effect	Industrial Maturity	Major Limitations
Conventional solvent extraction	Dissolution with aqueous-organic solvents	~22% (industrial average) [20]	Poor	Very high (current benchmark)	High solvent consumption, co-extraction of impurities
Pressurized liquid extraction (PLE)	High-temperature, high-pressure solvent extraction	Up to 42.4% [22]	Good (with acid additives)	Moderate	High initial equipment investment
Supercritical fluid extraction (SFE)	CO_2_-based extraction with polar modifiers	Up to 39.4% [22]	Good	Emerging	High pressure requirement, limited polarity range
Adsorption resin purification	Selective binding on macroporous resins	30–40% (from crude extract) [108]	Excellent	High	Adsorbent regeneration cost
Molecularly imprinted polymers (MIP)	Structure-specific recognition binding	—	Excellent	Laboratory scale	High preparation cost
Enzyme-assisted extraction	Proteolytic digestion of biomass matrix	~30% enhancement over conventional [110]	Excellent	Moderate	Long processing time

## Data Availability

Data sharing is not applicable to this article as no data were created or analysed in this study.
